# Direct and Trait‐Mediated Effects of Climate on Grassland Productivity Across the Alps

**DOI:** 10.1111/gcb.71142

**Published:** 2026-10-16

**Authors:** Gaüzère Pierre, Maria Hällfors, Jussi Mäkinen, Philippe Choler, Risto K. Heikkinen, Romain Goury, Joaquim Estopinan, Cyrille Violle, Franziska Schrodt, Sara Si Moussi, Mickaël Hedde, Luca Santini, Wilfried Thuiller

**Affiliations:** ^1^ Univ. Grenoble Alpes, Univ. Savoie Mont Blanc, CNRS, LECA Grenoble France; ^2^ Finnish Environment Institute Helsinki Finland; ^3^ CEFE, Univ Montpellier, CNRS, EPHE, IRD, INRAE Montpellier France; ^4^ School of Geography University of Nottingham Nottingham UK; ^5^ Eco&Sols, INRAE, IRD, CIRAD, Institut Agro, University Montpellier Montpellier France; ^6^ Department of Biology and Biotechnologies “Charles Darwin” Sapienza University of Rome Rome Italy

**Keywords:** alpine grasslands, biodiversity–ecosystem functioning, causal inference, climate variability, ecosystem productivity, environmental heterogeneity, functional traits, remote sensing

## Abstract

Understanding how climate shapes ecosystem productivity through energetic constraints and changes in the functional properties expressed by vegetation remains challenging. This is central to trait‐based biodiversity–ecosystem functioning research in mountain grasslands where warming and environmental gradients interact. Here, we distinguish direct climatic controls on productivity from indirect pathways operating through changes in canopy functional composition, physiological state, and within‐plot spectral functional heterogeneity across the European Alps. Using Sentinel‐2 time series (2017–2024), we quantified canopy functional structure from spectral proxies of pigment investment and water status, within‐plot spectral functional heterogeneity using the functional richness (FRic) metric, and vegetation productivity using near‐infrared reflectance of vegetation (NIRv). We combined panel‐data models with piecewise structural equation modeling to partition climate associations into direct and trait‐mediated components operating through canopy functional properties and used varying‐coefficient models to evaluate how these pathways vary along environmental gradients. We identified two pathways linking climate to productivity. Warmer growing seasons increased productivity directly but were associated with a lower chlorophyll‐to‐carotenoid pigment balance, consistent with greater relative carotenoid investment, generating an opposing indirect pathway. Greater winter snow accumulation reduced productivity primarily through a direct pathway, while its canopy‐mediated pathways were uncertain. Climatic water deficit reduced canopy water status and within‐plot spectral functional heterogeneity, but its direct and total associations with productivity were not distinguishable from zero. Environmental context modulated climate–vegetation relationships. Baseline temperature and moisture availability strongly shaped climate effects on canopy traits and productivity, whereas canopy‐property–productivity relationships varied little across space. Positive productivity responses to warming were stronger in wetter and more seasonally variable climatic contexts, but weakened where baseline winter snow accumulation was greater. Together, our findings show that short‐term productivity responses in mountain grasslands reflect a balance between direct energetic constraints and indirect pathways through integrated canopy functional properties, and that this balance varies along macroclimatic gradients.

## Graphical abstract

Climate change influences ecosystem productivity through two distinct but interacting pathways. First, climatic variability directly constrains photosynthetic activity via temperature and water limitation. Second, climate reshapes community functional composition, altering trait distributions that regulate ecosystem functioning. Using alpine grasslands as a model system, we integrate satellite‐derived productivity time series with trait‐based metrics to disentangle these pathways. Our results demonstrate that trait‐mediated effects substantially modulate productivity responses to climate variability. This approach advances a mechanistic understanding of how biodiversity structures ecosystem resilience under ongoing climatic change.

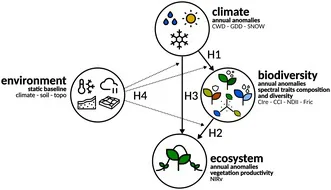

## RÉSUMÉ

Comprendre comment le climat façonne la productivité des écosystèmes à travers des contraintes énergétiques directes et des voies indirectes médiées par la biodiversité demeure un défi majeur en écologie du changement global, en particulier dans les prairies de montagne où le réchauffement rapide interagit avec de forts gradients environnementaux. Nous démêlons ici les effets climatiques directs sur la productivité des écosystèmes de leurs effets indirects opérant via la structure fonctionnelle de la canopée, à l'échelle de l'ensemble des Alpes européennes. À partir de séries temporelles Sentinel‐2 (2017–2024), nous avons quantifié la structure fonctionnelle de la canopée à partir de proxys spectraux liés aux pigments et à l'état hydrique, ainsi que la richesse fonctionnelle, et estimé la productivité à l'aide de la réflectance proche‐infrarouge de la végétation (NIRv). Nous avons combiné inférence causale et modèles d'équations structurelles pour partitionner les effets climatiques totaux en composantes directes et médiées par les traits, puis utilisé des modèles à coefficients variables pour analyser la modulation de ces voies le long des gradients environnementaux. Nos résultats montrent que deux voies concomitantes relient le climat à la productivité. Les saisons de croissance plus chaudes augmentent directement la productivité, tout en réduisant l'investissement pigmentaire et l'état hydrique de la canopée, générant des effets indirects négatifs qui atténuent partiellement les gains directs. Une accumulation neigeuse plus importante diminue la productivité à la fois directement, en raccourcissant la saison de croissance effective, et indirectement via des augmentations de l'eau dans la canopée associées à une productivité plus faible. Le déficit hydrique climatique produit des effets opposés: des impacts directs légèrement positifs compensés par des effets indirects négatifs médiés par les pigments, aboutissant à un effet net faible. La diversité fonctionnelle contribue modérément à ces médiations. Le contexte environnemental module fortement ces relations climat–végétation. La température et l'humidité de fond déterminent la variabilité spatiale des réponses, renforçant les gains sous réchauffement dans les zones humides de moyenne altitude et les atténuant aux hautes altitudes, dans les secteurs secs ou riches en neige. Ensemble, ces résultats montrent que les réponses de productivité résultent d'un équilibre entre contraintes énergétiques directes et ajustements physiologiques médiés par la biodiversité.

## 1 Introduction

Understanding how ecosystem functioning emerges from the interplay between abiotic constraints and biotic organization is a central challenge in ecology (Chapin 3rd et al. 2000). Climate sets fundamental limits on biological activity by controlling energy, water availability, and seasonality. Yet, ecosystems often respond to climatic variation in ways that cannot be explained solely by direct climatic effects (Lavorel and Garnier 2002; Suding et al. 2008). Instead, biodiversity—through the composition, structure, and diversity of species and their functional traits—can modulate, buffer, or amplify climate effects on ecosystem functioning (Chapin 3rd et al. 2000; de Bello et al. 2021; Dee et al. 2023; Lavorel and Garnier 2002). However, the relative contributions of direct climatic controls and indirect pathways through vegetation functional properties remain unresolved. How this balance varies across broad environmental gradients is particularly uncertain. Mountain grasslands provide a relevant system in which to address this question. Along steep environmental gradients, productivity may shift from energy‐limited to water‐limited regimes (Denissen et al. 2022), while plant communities simultaneously reorganize in terms of dominant strategies and functional diversity. In such systems, climate change may influence productivity directly, for example through temperature‐driven changes in growing‐season length or snow dynamics (Körner 2021a; Piao et al. 2011; Wipf and Rixen 2010), and indirectly through shifts in trait composition and functional diversity that alter community‐level resource use (Lavorel and Garnier 2002; Suding et al. 2008). Mountain plant communities may also respond to changing climatic conditions with substantial temporal lags, while increasingly frequent warm and dry conditions can alter canopy functioning and productivity before major compositional changes become apparent (Alexander et al. 2018; Block et al. 2022; Choler 2023; Corona‐Lozada et al. 2019; Visakorpi et al. 2023).

Biodiversity and ecosystem responses to climate change can be constrained by local environmental conditions, so that similar climatic anomalies produce contrasting ecological outcomes depending on the baseline abiotic context (Mod et al. 2016; Suding et al. 2008). Biodiversity–functioning relationships likely depend on local differences in abiotic constraints, resource availability, and community composition (Leibold et al. 2017). Mapping such spatial heterogeneity can reveal under which conditions biodiversity and ecosystem functioning are most sensitive to climate change, and where trait composition and diversity most strongly impact ecosystem functioning. In addition, revealing this heterogeneity helps identify where conservation or management interventions may be most effective. Yet, despite decades of research on biodiversity–ecosystem functioning, we still lack spatially explicit estimates of how much short‐term variation in ecosystem productivity is associated directly with climate versus indirectly with functional properties expressed by vegetation, and how this balance varies across environmental contexts in natural systems.

Within the biodiversity–ecosystem functioning framework, several non‐exclusive mechanisms have been proposed. Ecosystem functioning can be driven by the traits of dominant species, functional complementarity among species, and response diversity, with recent work emphasizing that these mechanisms arise from the temporal dynamics of trait distributions, species abundances, and environmental filtering in naturally assembled communities (De Bello 2021; Dee et al. 2023; Grime 1998; Loreau and Hector 2001). Trait Driver Theory (TDT) formalizes this view by predicting that ecosystem functioning is governed by the distribution of trait values expressed within communities, with directional selection toward phenotypes maximizing resource use efficiency under given environmental constraints (Enquist et al. 2015). While these mechanisms are experimentally well‐supported, their relative importance in shaping productivity across natural landscapes remains debated. Translating these mechanisms from experimental settings to heterogeneous natural landscapes requires observations that capture how functional strategies are expressed across space and time.

Recent advances in optical remote sensing allow vegetation functional properties to be monitored continuously across large spatial extents (Cavender‐Bares, Schneider, et al. 2022; Ustin and Gamon 2010).

Leaf pigments, water status and canopy structure leave consistent spectral signatures that reflect plant physiological strategies and responses to stress, and recent studies have demonstrated the potential of optical remote sensing to quantify these properties across spatial scales (Ali et al. 2020; Cavender‐Bares, Gamon, and Hobbie 2022; Cavender‐Bares, Schneider, et al. 2022; Damm et al. 2018). Spectral indices derived from multispectral imagery therefore provide integrative proxies of realized canopy functional structure and physiological state, capturing both functional dominance and within‐species physiological adjustment. When combining climatic time series with such proxies of vegetation functional state and ecosystem productivity, it is possible to investigate climate–canopy–ecosystem functioning relationships within a broader biodiversity–ecosystem functioning framework beyond experimental plots and across large extents and heterogeneous landscapes. However, the volume and dimensionality of remote‐sensing time series also pose a challenge: patterns derived from thousands of plots and multiple correlated indices are potentially confounded by shared physiological processes. Panel‐data and mediation approaches can help distinguish direct climatic associations from indirect trait‐mediated pathways under explicit assumptions, while recognizing the possibility of shared environmental forcing (Van Cleemput et al. 2025).

Here, we distinguish two dimensions of realized canopy functional organization. CIre, CCI and NDII are community‐integrated, CWM‐like indicators of canopy pigment and water status that may vary through physiological or phenological plasticity and shifts in the relative dominance of species or functional groups. By contrast, the FRic metric quantifies the breadth of pixel‐integrated canopy functional states among Sentinel‐2 pixels within each plot. Because each pixel integrates multiple individuals and potentially multiple species, we interpret FRic as within‐plot spectral functional heterogeneity rather than individual‐ or species‐level alpha functional diversity. Biologically, it is conceptually closer to sub‐plot beta functional diversity (Rossi et al. 2020; Zhao et al. 2025). We therefore do not interpret interannual variation in these metrics as direct evidence of taxonomic turnover, but retain their relevance to the broader trait‐based biodiversity–ecosystem functioning framework. However, disentangling their roles requires separating direct climatic effects (e.g., through energy or water limitation) from indirect pathways through canopy functional properties, which remain difficult to distinguish across spatial and temporal gradients in natural systems (Dee et al. 2023; van der Plas 2019).

To address these challenges and explicitly link the theoretical expectations above to spatially explicit data, we combine metrics derived from remote‐sensing time series with panel‐data and mediation modeling approaches (Dee et al. 2023; Ferraro et al. 2019). This allows us to estimate direct and indirect pathways linking climate variability, canopy functional dynamics, and ecosystem productivity under the stated identification assumptions. We measure climate variability through interannual anomalies in temperature, water, and snow; canopy functional dynamics through community‐integrated spectral proxies of canopy pigment investment and water status, together with within‐plot spectral functional heterogeneity quantified using FRic; and ecosystem productivity by integrating NIRv across the growing season (Cavender‐Bares, Gamon, and Hobbie 2022; Badgley et al. 2017). We test four inter‐linked hypotheses (Figure 1).

- (H1) Interannual climatic anomalies modify canopy functional structure, reflected in spectral trait composition and within‐plot spectral functional heterogeneity, through stress filtering and physiological adjustment.
- (H2) Variation in canopy functional structure is associated with ecosystem productivity, with community‐integrated pigment‐ and water‐related properties expected to show stronger associations than within‐plot spectral functional heterogeneity, consistent with mass‐ratio and trait‐driver expectations.
- (H3) Climate additionally affects productivity directly through energy and water limitation, with warmer growing seasons expected to enhance productivity, greater snow accumulation and higher water deficit to reduce it.
- (H4) The strength and direction of all pathways can be mediated by background environmental conditions, such as average climate, soil conditions, and topography.

**FIGURE 1:**
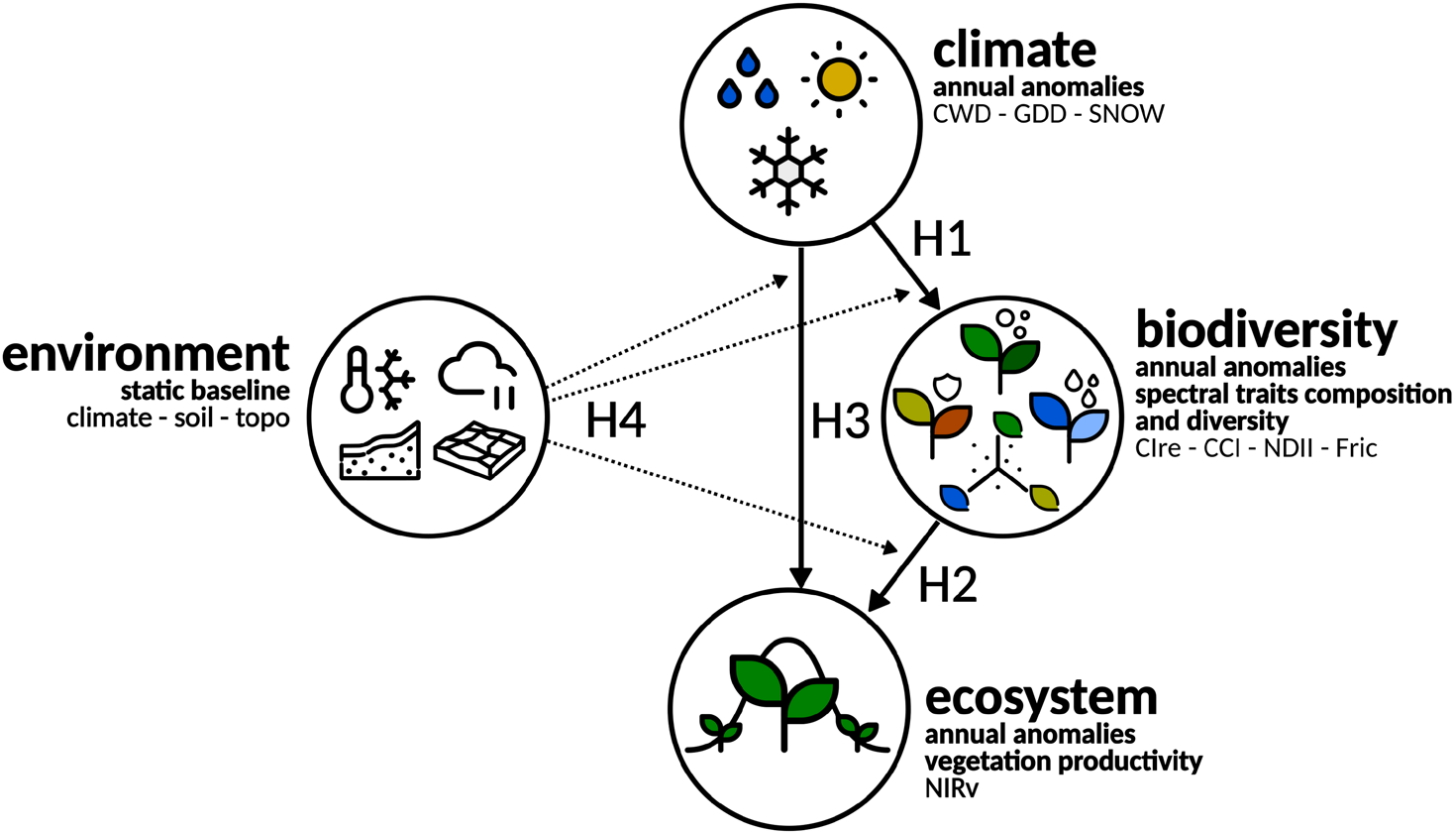
Conceptual pathways linking climate anomalies, canopy functional properties and ecosystem productivity, including study hypotheses (H). (H1) Annual climatic anomalies are associated with community‐integrated indicators of canopy pigment and water status (CIre, CCI and NDII) and with within‐plot spectral functional heterogeneity quantified using FRic. CIre is a red‐edge chlorophyll index describing chlorophyll‐related canopy state and potential photosynthetic capacity; CCI is a chlorophyll–carotenoid index sensitive to the relative balance between chlorophyll and carotenoid pigments; NDII is a near‐infrared index related to canopy water status; and FRic quantifies the breadth of pixel‐integrated canopy functional states among Sentinel‐2 pixels within a plot and is interpreted as spatial functional heterogeneity rather than individual‐ or species‐level alpha functional diversity. (H2) Variation in these canopy properties is conditionally associated with ecosystem productivity, measured using near‐infrared reflectance of vegetation (NIRv). (H3) Climate is also associated directly with productivity through energy and water limitation. (H4) The estimated pathways may vary with baseline climate, soil and topography. Solid arrows indicate modeled pathways, whereas dashed arrows indicate environmental moderation. Hypotheses are evaluated using panel‐data and mediation models, with detailed mechanistic expectations in Supporting Information Text S1. Although CIre and NIRv both use near‐infrared reflectance, CIre is a red‐edge ratio describing chlorophyll‐related canopy state, whereas NIRv combines NDVI with near‐infrared reflectance and is integrated across the growing season as a proxy for annual vegetation productivity.

Directional and mechanistic expectations associated with each hypothesis are detailed in Supporting Information S1. To test these predictions across natural grasslands of the Alps, we quantified how annual climatic anomalies translated into anomalies of canopy functional structure and productivity. We then partitioned total climate effects on productivity into (1) direct and (2) indirect trait‐mediated components using piecewise Structural Equation Models (SEMs). Finally, we mapped how these pathways vary along background gradients in climate, soil, and topography. This analytical framework extends trait‐based biodiversity–ecosystem functioning questions to large spatial scales while distinguishing explicitly between dominance‐related canopy properties, within‐plot spectral functional heterogeneity, and abiotic effects.

## 2 Methods

### 2.1 Study Area and Sampling Design

We focused on natural grasslands located above 1000 m a.s.l. across the European Alps, a system characterized by strong climatic gradients, short growing seasons, and relatively homogeneous low‐intensity land use. Mountain grasslands are predominantly managed through traditional summer grazing, with limited fertilization or irrigation, allowing vegetation dynamics to be interpreted primarily in relation to climatic variability rather than abrupt land‐use change. Using land‐cover and elevation data, we delineated a continuous grassland mask across the Alpine arc and randomly selected 10,000 plot centroids within this area. Each plot was defined as a circular buffer with a 60 m radius—a spatial scale chosen to integrate multiple Sentinel‐2 pixels while being small enough to capture relatively homogeneous grassland conditions. All analyses were conducted at the plot level. Detailed geospatial processing steps are provided in the Supporting Information Text S3.

We defined our study area as natural grasslands located above 1000 m asl within the European Alps and delineated it in Google Earth Engine. First, we loaded CORINE Land Cover 2018 (100 m) data and retained pixels classified as “Natural grasslands” (Class 3.2.1). We used elevation obtained from the Copernicus GLO30 Digital Elevation Model (DEM) thresholded at > 1000 m. To isolate the Alps, we intersected these pixels with a hand‐drawn polygon approximating the Alpine arc. The “grassland” and “elevation” masks were combined and clipped to this polygon to define the study area. To avoid border effects (i.e., ecotones and minor boundary errors), we further eroded the borders of the resulting mask by removing a 100 m inward buffer, thereby excluding edge pixels and ensuring that the subsequent 60 m plot buffers remained fully within the grassland patches. To sample analysis points, we drew a random sample of 10,000 points within this study area and defined each point as the center of a geodesic circular polygon with a 60‐m radius, hereafter referred to as “plot”.

### 2.2 Remotely Sensed Vegetation Functioning and Canopy Functional Properties

We characterized interannual variation in vegetation functioning and canopy properties using satellite observations from 2017–2024. All vegetation metrics were derived from Sentinel‐2 surface reflectance imagery at 10–20 m spatial resolution and processed consistently across years. Analyses were restricted to the growing season (April–October), and standard quality masks were applied to remove clouds, snow, and ice. We retained only pixel observations with fractional vegetation cover (FVC) ≥ 0.60, thereby excluding rocky, sparsely vegetated, or mixed surfaces known to bias pigment‐ and water‐related indices. This filtering improves the reliability of vegetation‐property estimates in heterogeneous alpine terrain by ensuring that vegetation remains the dominant component of the spectral signal. Because the FVC threshold was applied at the observation level, low‐cover states within otherwise included grassland plots may be underrepresented, particularly at sparse sites or during years with reduced vegetation cover. Our inference is therefore conditional on sufficiently vegetated observations and does not extend to very sparse grassland–rock mosaics. Plot‐level values were obtained by aggregating all retained pixels within each plot.

### 2.3 Canopy Functional Composition and Within‐Plot Spectral Functional Heterogeneity

We used three spectral indices to characterize canopy functional structure: the red‐edge chlorophyll index (CIre) related to canopy chlorophyll content and potential photosynthetic capacity (Ali et al. 2020; Clevers and Gitelson 2013; Croft et al. 2017); the chlorophyll–carotenoid index (CCI), sensitive to the chlorophyll‐to‐carotenoid pigment balance and photosynthetic phenology (Gamon et al. 2016; Helfenstein et al. 2022, 2025); and the normalized difference infrared index (NDII), related to canopy water status and drought sensitivity (Damm et al. 2018; Hardisky et al. 1983). CIre, CCI, and NDII were represented by the 80th percentile of valid April–October observations to capture near‐peak canopy functional states while reducing sensitivity to residual noise, extreme observations, and early‐ or late‐season phenological conditions. These indices capture complementary aspects of plant physiological functioning, including photosynthetic pigment investment, sustained photoprotective pigment balance, and canopy water status. For CCI, higher values indicate a higher chlorophyll‐to‐carotenoid balance associated with greater photosynthetic activity, whereas lower values indicate relatively greater carotenoid investment, consistent with increased sustained photoprotection or stress acclimation. Instead of interpreting these indices as direct measurements of taxonomic or trait composition, we treat them as proxies of realized canopy functional structure, integrating the functional properties expressed by the plant community in a given year. Variation in these indices can arise from shifts in species dominance (mass‐ratio effects) and/or within‐species physiological and phenotypic adjustment to interannual climatic variability. Consequently, the indirect pathways estimated in our analyses should be interpreted as operating through integrated canopy functional properties rather than through taxonomic turnover. To quantify within‐plot spectral functional heterogeneity, we applied the functional richness (FRic) metric to the multivariate spectral trait values of all retained Sentinel‐2 pixels within each plot‐year. FRic therefore measures the extent of the spectral trait space occupied by pixel‐integrated canopy states within a plot. Because each pixel integrates the canopy signal of multiple individuals and potentially multiple species, variation among pixels should not be interpreted as individual‐ or species‐level alpha functional diversity. Instead, the metric captures spatial heterogeneity among community‐integrated canopy states at sub‐plot scales and is therefore conceptually closer to beta functional diversity, as emphasized for Sentinel‐2 grassland applications by Rossi et al. (2020) and Zhao et al. (2025). We refer to this metric throughout as within‐plot spectral functional heterogeneity (FRic).

### 2.4 Ecosystem Productivity

Ecosystem productivity was quantified using near‐infrared reflectance of vegetation (NIRv), a satellite‐based proxy of gross primary productivity that integrates canopy structure, light absorption, and photosynthetic activity (Badgley et al. 2017). NIRv was calculated for each valid Sentinel‐2 acquisition between April and October, summed across acquisitions for each pixel to approximate cumulative growing‐season productivity, and subsequently summarized at the plot level.

Because climate influences integrated canopy properties that jointly affect multiple spectral bands, spectral traits and NIRv are expected to covary to some extent: they describe complementary but not structurally independent dimensions of canopy functioning. In our causal framework, this shared variance is interpreted as an ecologically meaningful signal rather than redundant radiometric information. Importantly, spectral traits summarize near‐peak canopy physiological state, whereas productivity integrates vegetation functioning over the full growing season, ensuring that mediators and outcomes capture related but distinct aspects of ecosystem functioning. We therefore did not orthogonalize spectral traits against NIRv, as doing so would remove the specific biophysical signal through which climate is hypothesized to affect productivity via canopy functional structure. However, CIre, CCI, and NDII plot‐level values may vary with vegetation amount and canopy structure. Likewise, NIRv integrates photosynthetic functioning with the amount and spatial distribution of vegetation. The pathways estimated below should therefore be interpreted as operating through integrated canopy functional properties rather than as effects of leaf‐level physiology independent of canopy structure.

### 2.5 Climate Variability and Environmental Context

We characterized interannual climatic variability using three variables representing the primary abiotic constraints on mountain grassland functioning: thermal energy availability, water limitation, and snow dynamics. Thermal conditions were quantified using growing degree days above 5°C accumulated during the April–October growing season, and water limitation using climatic water deficit over the same growing‐season period. Snow dynamics were represented by the maximum winter snow water equivalent preceding each growing season. Thus, GDD and CWD characterize growing‐season conditions, whereas SWE captures antecedent winter snowpack. These variables were selected based on well‐established theoretical and empirical links between climate, plant physiological activity, and growing‐season length in mountain ecosystems. Climate time series were extracted for each plot for the period 2017–2024 from gridded reanalysis and climatological datasets. Although the native spatial resolution of these products is coarser than the plot resolution, our inference targets within‐plot interannual anomalies rather than fine‐scale spatial gradients rendering this approach suitable. Growing degree days were derived from hourly ERA5‐Land data at approximately 9 km resolution, while climatic water deficit and snow water equivalent were derived from monthly TerraClimate data at approximately 4 km resolution. Climate variables should therefore be interpreted as year‐to‐year deviations experienced in each plot relative to its mean climatic context, rather than as precise microclimatic estimates.

To characterize the background environmental context, we compiled a set of static environmental variables describing long‐term climatic conditions, soil properties, and topography harmonized across a continuous 100 × 100 m grid covering the study area (Figures 2 and 3). These variables primarily vary across space and are assumed to remain constant over the study period. They were used as effect modifiers to test whether the strength and direction of climate–canopy–productivity relationships vary along environmental gradients, rather than as direct drivers of vegetation dynamics.

**FIGURE 2:**
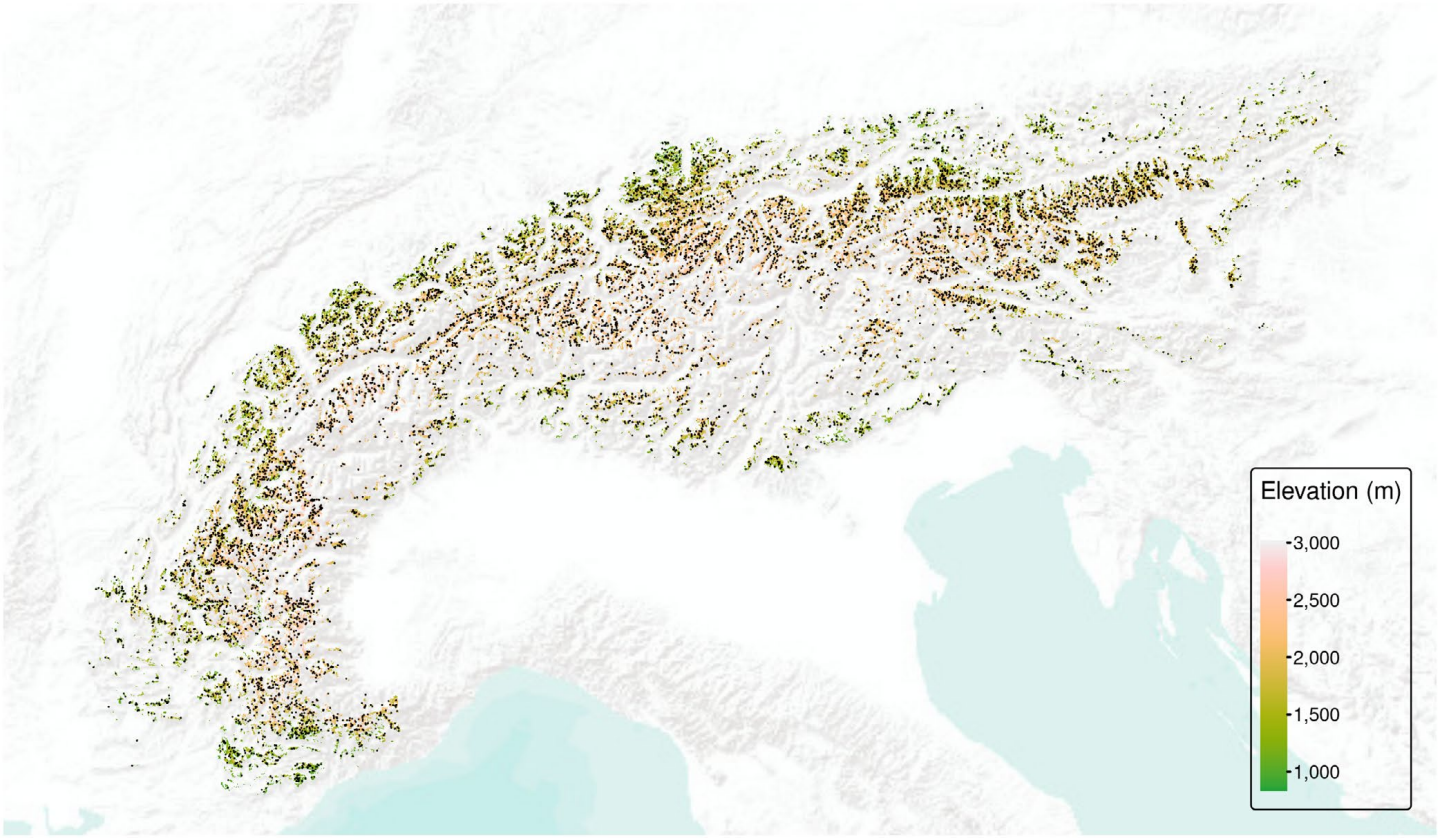
Study area and sampling design. Natural grasslands (identified based on CORINE Land Cover 2018, Class 3.2.1) located above 1000 m across the Alpine arc visualized over a shaded‐relief background. Black points mark randomly selected plot centroids, around which 60‐m‐radius circular plots were defined (buffers not displayed to enhance visibility).

**FIGURE 3:**
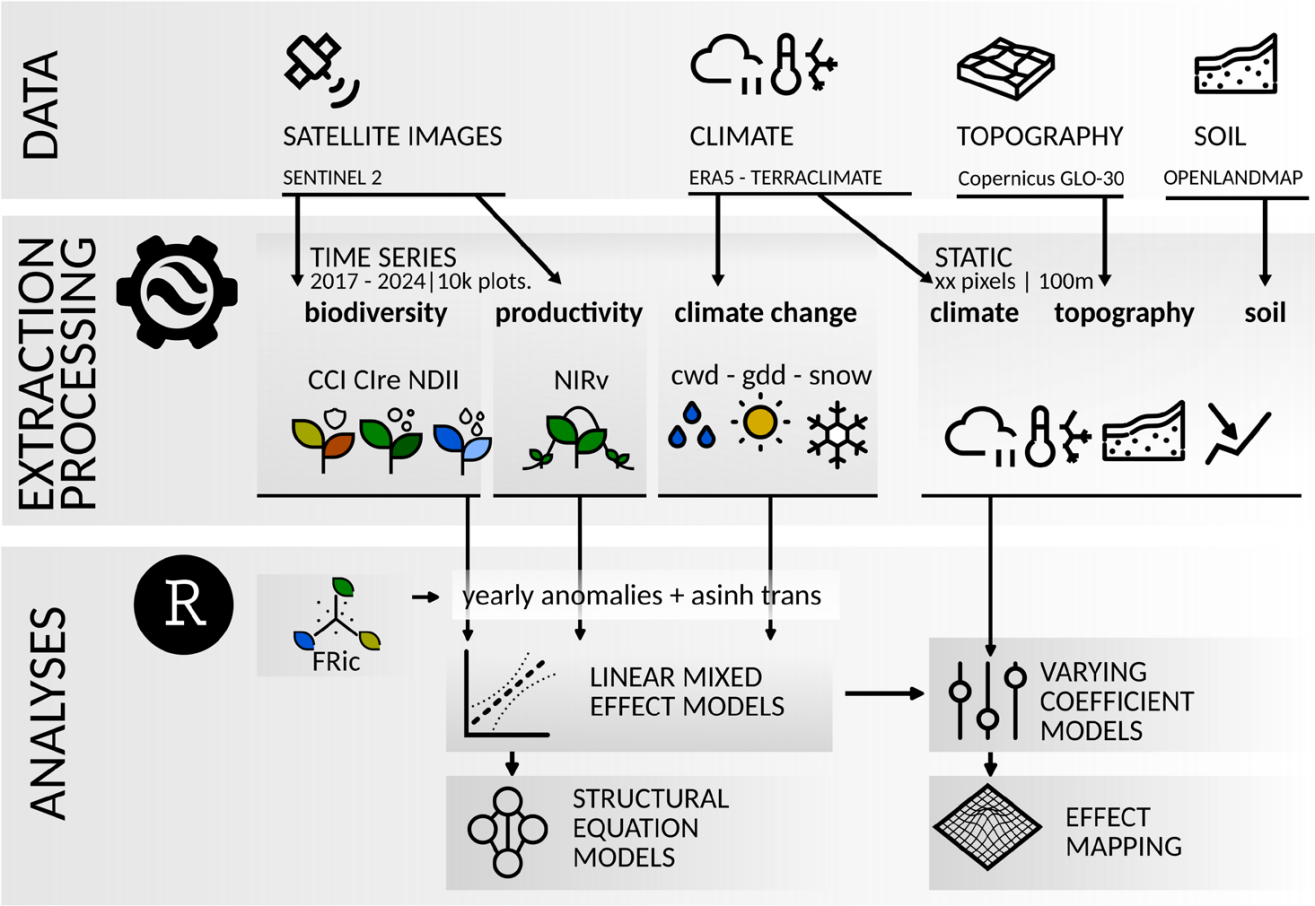
Analysis workflow. From satellite (Sentinel‐2) and climate reanalyses (era5/terraclimate), we derived annual time series (2017–2024~10,000 plots) of spectral‐trait composition (CCI, CIre, NDII), within‐plot spectral functional heterogeneity (FRic), and productivity (NIRv), together with interannual climate anomalies (GDD, cwd, swe) and static environment grids (climate, topography from copernicus glo‐30, and soils from openlandmap). Time‐varying variables were converted to within‐plot yearly anomalies and transformed with inverse hyperbolic functions (asinh). Inference used linear mixed‐effects models with plot and year effects to estimate within‐plot responses. Piecewise structural equation models combined these fits to separate direct climate effects on productivity from indirect pathways via canopy functional properties. Spatial heterogeneity was assessed with varying‐coefficient LMMs, where climate slopes vary by baseline environment. The fitted effects were projected over the alpine raster stack to produce effect and uncertainty maps.

### 2.6 Study Design and Causal Inference Framework

#### 2.6.1 Estimating Within‐Plot Pathways

Our analyses targeted how year‐to‐year climatic anomalies influence canopy functional structure and, in turn, ecosystem productivity. We addressed three linked questions: (i) how climate affects canopy functional structure and within‐plot spectral heterogeneity (H1), (ii) how canopy functional structure is associated with productivity (H2), and (iii) how climate affects productivity directly and indirectly through trait‐mediated pathways (H3). To isolate within‐plot effects, all time‐varying variables were expressed as interannual anomalies by subtracting plot‐specific means. This design removed all time‐invariant differences among plots, such as persistent soil conditions or long‐term management, and focused inference on temporal climatic forcing. Models included plot‐level random effects to account for repeated measurements and year fixed effects to absorb shocks common to all plots in a given year. We used linear mixed models to estimate within‐plot relationships among climate, canopy functional structure, and productivity. Outcomes and predictors were analyzed on an inverse hyperbolic sine scale, which stabilizes variance and accommodates zero and negative anomaly values. As a robustness check, core models were re‐estimated using two‐way fixed‐effects regressions, yielding consistent results (Supporting Information Text S4). It must be noted that because spectral canopy properties and productivity were summarized over overlapping portions of the same growing season, the annual data do not resolve their precise within‐season temporal ordering. The indirect paths therefore quantify relationships consistent with climate‐driven changes in canopy functional states contributing to seasonally integrated productivity, rather than demonstrating a strict temporal sequence among within‐season processes.

#### 2.6.2 Partitioning Direct and Trait‐Mediated Climate Effects

To separate direct climatic effects on productivity from indirect effects operating through canopy functional structure, we linked the fitted mixed models into a piecewise structural equation framework. Climate–canopy models quantify how climatic anomalies modify canopy functional composition and within‐plot spectral functional heterogeneity, while productivity models including both climate and canopy functional properties quantify the conditional associations between these properties and variation in NIRv. For each climatic driver, the total effect on productivity was decomposed into a direct component and indirect components operating through the measured canopy functional properties, including FRic‐based within‐plot spectral heterogeneity.

#### 2.6.3 Residual Spatial Autocorrelation

Residual spatial autocorrelation was assessed from conditional model residuals by calculating Moran's I separately within each year across eight distance classes. Residual dependence was strongest below 10 km and declined to approximately zero from 150 km onward. We therefore based final inference on 95% percentile intervals from a 150‐km spatial block bootstrap with 1000 replicates, retaining each plot's complete temporal sequence within resampled blocks. Direct, indirect, and total effects were recalculated within each of the 1000 150‐km spatial‐block bootstrap replicates using the same block resample across component models, and statistical support was based on the corresponding 95% bootstrap percentile intervals. Full diagnostics, sensitivity analyses, and resampling details are provided in Supporting Information Text S3.

#### 2.6.4 Spatial Heterogeneity in Climate Effects

To assess how climate impacts vary across environmental contexts, we fitted varying‐coefficient mixed models allowing within‐plot climate effects to depend linearly on static environmental variables describing average climate, soils, and topography. These models quantify how background environmental conditions modulate climate–canopy and climate–productivity relationships. Using these models, we mapped spatial variation in local climate effects across the Alpine grasslands, restricting predictions to environmental conditions represented in the data. Uncertainty in mapped effects was quantified by propagating parameter uncertainty from the fitted models. Additional methodological details, including data sources, preprocessing steps, statistical formulations, robustness checks, and uncertainty propagation, are provided in (Supporting Information Text S3).

## 3 Results

### 3.1 Direct and Trait‐Mediated Effects of Climate Anomalies on Grassland Productivity

Across the Alps, interannual climate variability affected productivity through a direct pathway and an indirect pathway mediated by canopy functional structure. Higher growing‐season thermal accumulation (GDD) had a positive direct effect on productivity and a negative effect on CCI. Greater winter snow accumulation (SWE) had a negative direct effect on productivity, but its canopy‐mediated pathways were uncertain. Climatic water deficit (CWD) reduced NDII and within‐plot spectral functional heterogeneity (FRic), but its direct and total effects on productivity were not supported. Unless otherwise stated, statistical support in the following sections refers to 95% bootstrap percentile intervals obtained from the 150‐km spatial block bootstrap.

*Spatial‐bootstrap inference from mixed models supported three climate effects on canopy properties (Figure* 4, *left panel). CCI declined with higher growing‐season thermal accumulation (GDD), indicating a lower chlorophyll‐to‐carotenoid pigment balance and relatively greater carotenoid investment, NDII declined with higher climatic water deficit (CWD), and within‐plot spectral functional heterogeneity (FRic) declined with higher CWD. The remaining climate–canopy associations, including all effects on CIre and the effects of winter snow water equivalent (SWE) on canopy properties, had* 95*% intervals overlapping zero*.

*Vegetation productivity, quantified using growing‐season NIRv, varied consistently with canopy spectral properties (Figure* 4, *top‐right panel). NIRv increased with higher CIre and CCI and decreased with higher NDII and within‐plot spectral functional heterogeneity (FRic)*.

*When accounting for canopy trait‐mediated pathways, NIRv increased with higher GDD and decreased with higher SWE, whereas the direct CWD effect had an interval overlapping zero (Figure* 4, *bottom‐right panel). CIre and CCI retained positive associations with NIRv, whereas NDII and FRic retained negative associations*.

**FIGURE 4:**
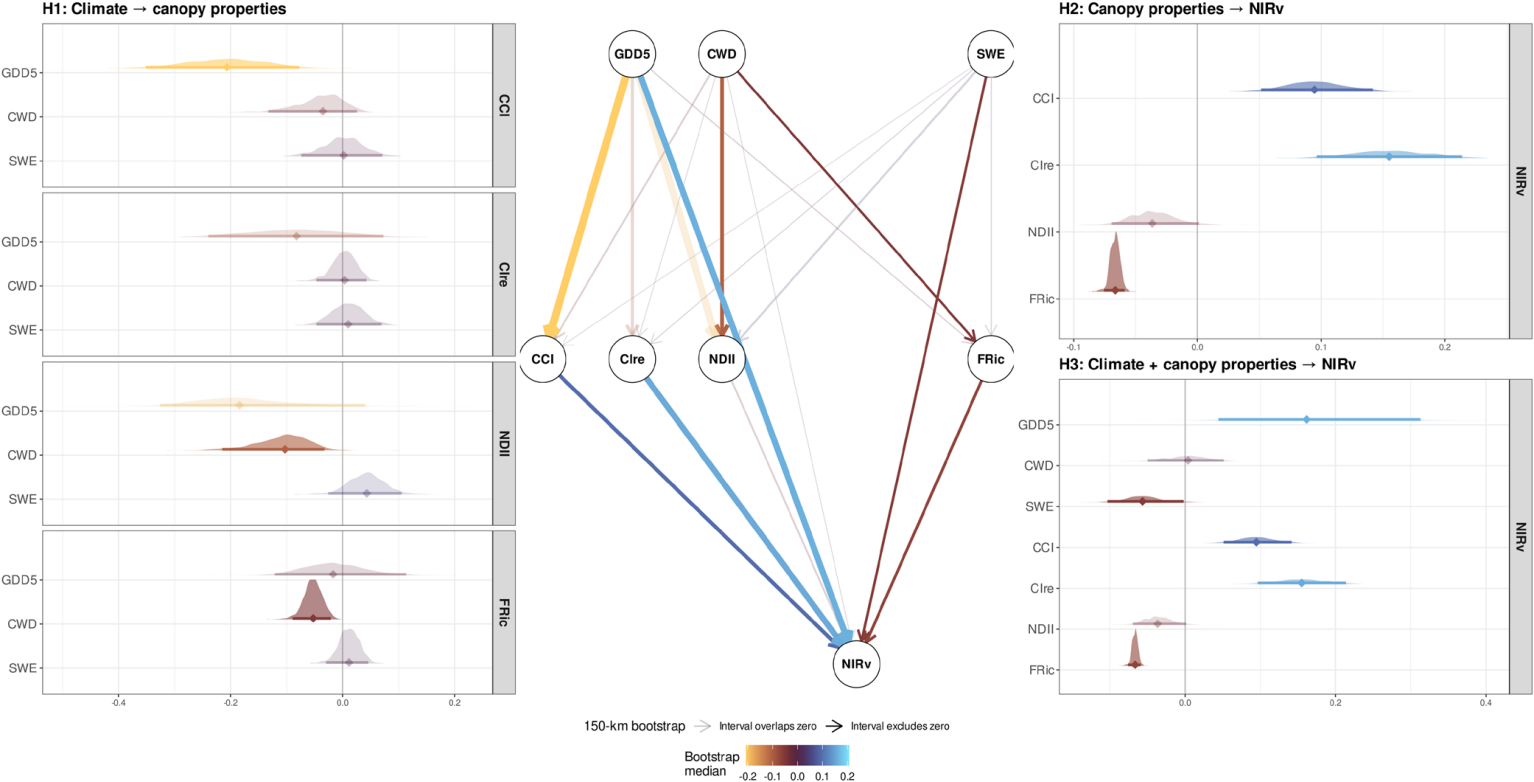
Within‐plot (demeaned) LMM effects and the corresponding piecewise SEM, with inference based on the 150‐km spatial block bootstrap. Left: H1 climate → canopy properties; top‐right: H2 canopy properties → productivity (NIRv); bottom‐right: H3 climate + canopy properties → NIRv. Ridge plots show the empirical distribution of coefficient estimates across the 1000 spatial‐block bootstrap replicates. Filled diamonds indicate bootstrap medians and thick horizontal intervals the 2.5th–97.5th percentile intervals; intervals overlapping zero indicate statistically unsupported relationships. The central path diagram summarizes the same relationships using bootstrap median coefficients: Edge color indicates effect direction and magnitude, line width scales with absolute effect size, and edge transparency distinguishes pathways for which the 95% bootstrap interval overlaps zero from those for which the interval excludes zero.

#### 3.1.1 Putting the Pathways Together: Structural Equation Model

The structural equation model identified two concurrent pathways linking interannual climate variability to ecosystem productivity (Figure 4, middle): an indirect pathway mediated by canopy spectral traits and a direct pathway from climate anomalies to productivity. The relative magnitudes of the direct, summed‐indirect, and total effects are summarized in Figure S5, while mediator‐specific indirect effects are shown in Figure S6.

For growing‐season thermal accumulation (GDD), the indirect pathway operated primarily through changes in pigment‐related traits. Higher GDD reduced CCI, and CCI was positively associated with NIRv. The CIre‐, NDII‐, and FRic‐mediated GDD effects and the summed indirect effect were uncertain. Nevertheless, the positive direct effect was larger, yielding a supported positive total effect of GDD on productivity. For winter snow water equivalent (SWE), the direct and total effects on NIRv were negative, but none of the individual or summed indirect pathways were supported. For climatic water deficit (CWD), the indirect point estimates through NDII and FRic were positive because CWD reduced both canopy properties, and both were negatively associated with NIRv; however, only the FRic‐mediated indirect effect had a 95% bootstrap interval excluding zero. The NDII‐, CCI‐, and CIre‐mediated effects, the direct effect, the summed indirect effect, and the total effect of CWD all had intervals overlapping zero.

### 3.2 Environmental Modulation and Spatial Variation of Effects

Environmental context modified several climate–canopy and climate–productivity pathways (Figure 5), with the strongest and most widespread moderation occurring for climate effects on canopy properties (H1). Baseline climatic variables generally produced the broadest variation in effect strength, while topographic conditions also strongly modified selected pathways, and soil effects were generally smaller.

**FIGURE 5:**
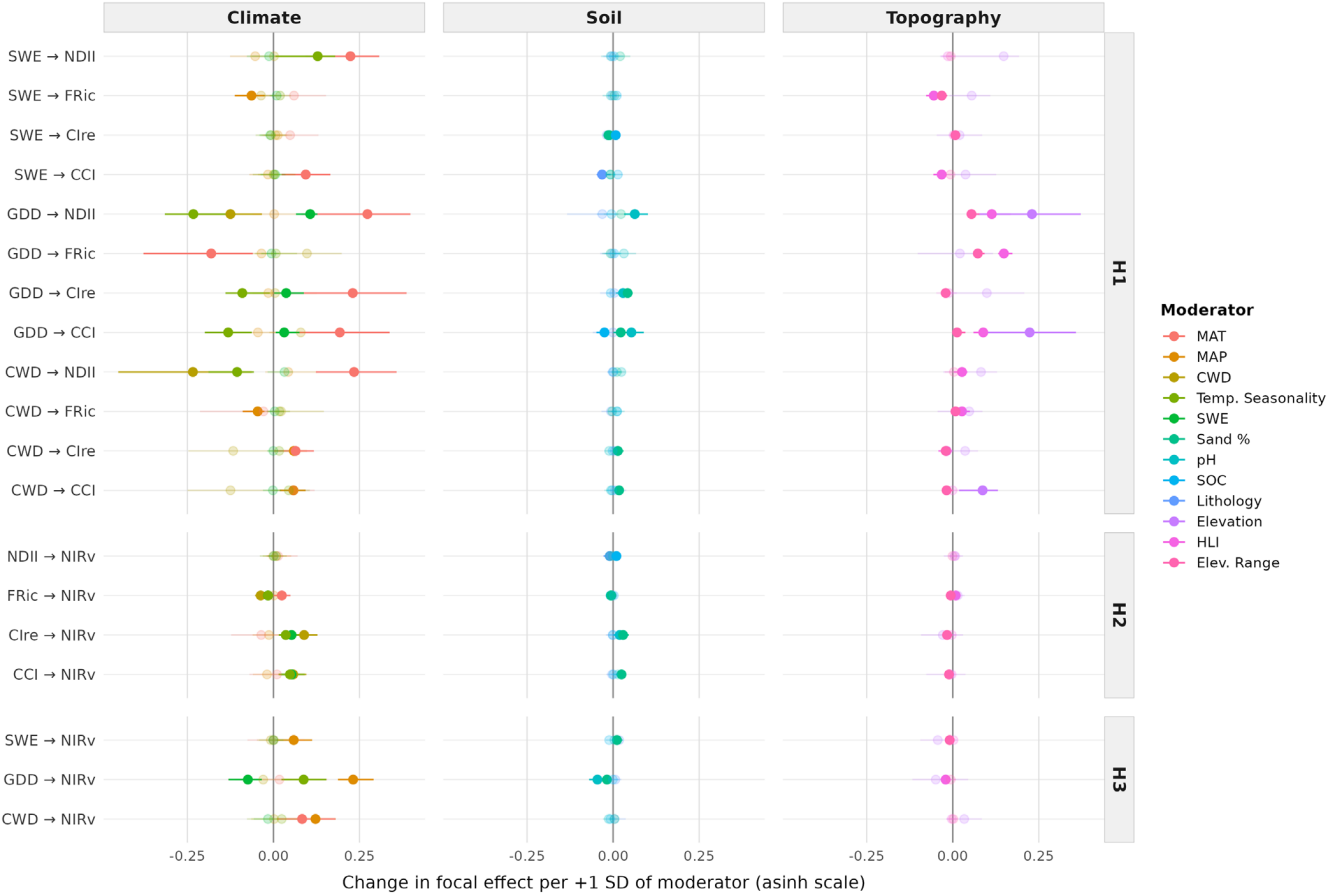
Environmental modulation of climate–canopy–productivity pathways across the Alps. Varying‐coefficient mixed models quantify how baseline climatic, soil and topographic conditions modify the focal within‐plot relationships corresponding to H1 (climate → canopy properties), H2 (canopy properties → productivity), and H3 (climate → productivity conditional on canopy properties). Points represent estimated moderator coefficients, expressed as the change in the focal‐effect slope per +1 SD increase in the environmental moderator, and horizontal lines show 95% percentile intervals from the 150‐km spatial block bootstrap. Colors identify individual environmental moderators. Estimates for which bootstrap intervals exclude zero are shown at full opacity, whereas estimates for which intervals overlap zero are faded. The vertical line indicates no moderation. All panels share the same *x*‐axis scale to facilitate comparison of moderator strength among climate, soil, and topographic variables.

In H1 (Figure 5, top panels), environmental modulation was strongest for the GDD → CCI and CWD → NDII relationships. The negative GDD → CCI relationship varied particularly with baseline thermal conditions and topography, becoming weaker with higher mean annual temperature and elevation but stronger with greater temperature seasonality. The negative CWD → NDII relationship strengthened toward sites characterized by greater long‐term water deficit and temperature seasonality, but weakened in warmer locations. Moderation of the CWD → within‐plot spectral functional heterogeneity (FRic) relationship was comparatively weaker.

In H2 (Figure 5, middle panels), environmental modulation was generally weaker. Effect sizes linking spectral traits and within‐plot spectral functional heterogeneity to NIRv showed limited variation across baseline climatic, soil, and topographic gradients.

In H3 (Figure 5, bottom panels), baseline climatic conditions were the dominant moderators of the GDD → NIRv relationship. The positive productivity response to growing‐season thermal accumulation strengthened with higher long‐term precipitation and temperature seasonality, but weakened with greater baseline winter snow accumulation. The CWD → NIRv relationship also varied with baseline climate despite its uncertain average effect, illustrating that weak average relationships can mask contrasting responses across environmental contexts.

#### 3.2.1 Focus on GDD → CCI Environmental Modulation

Baseline environmental conditions explained 19.7% of the plot‐to‐plot variation in the fitted GDD → CCI effect. Spatial‐bootstrap inference identified mean annual temperature, mean elevation, and temperature seasonality as the clearest moderators (Figure 5). The negative GDD → CCI relationship became weaker with higher mean annual temperature and elevation, but stronger with greater temperature seasonality. Other moderator estimates were smaller or had 95% bootstrap intervals overlapping zero and are therefore not interpreted as statistically supported effects. The spatial projection in Figure 6 illustrates the geographic pattern predicted by the full varying‐coefficient model, with stronger negative effects in parts of the northern and eastern Alps and weaker or locally neutral effects toward the south‐west. Because the map integrates all fitted moderators, including individually uncertain ones, it is interpreted descriptively rather than as evidence that every local spatial contrast is statistically supported.

**FIGURE 6:**
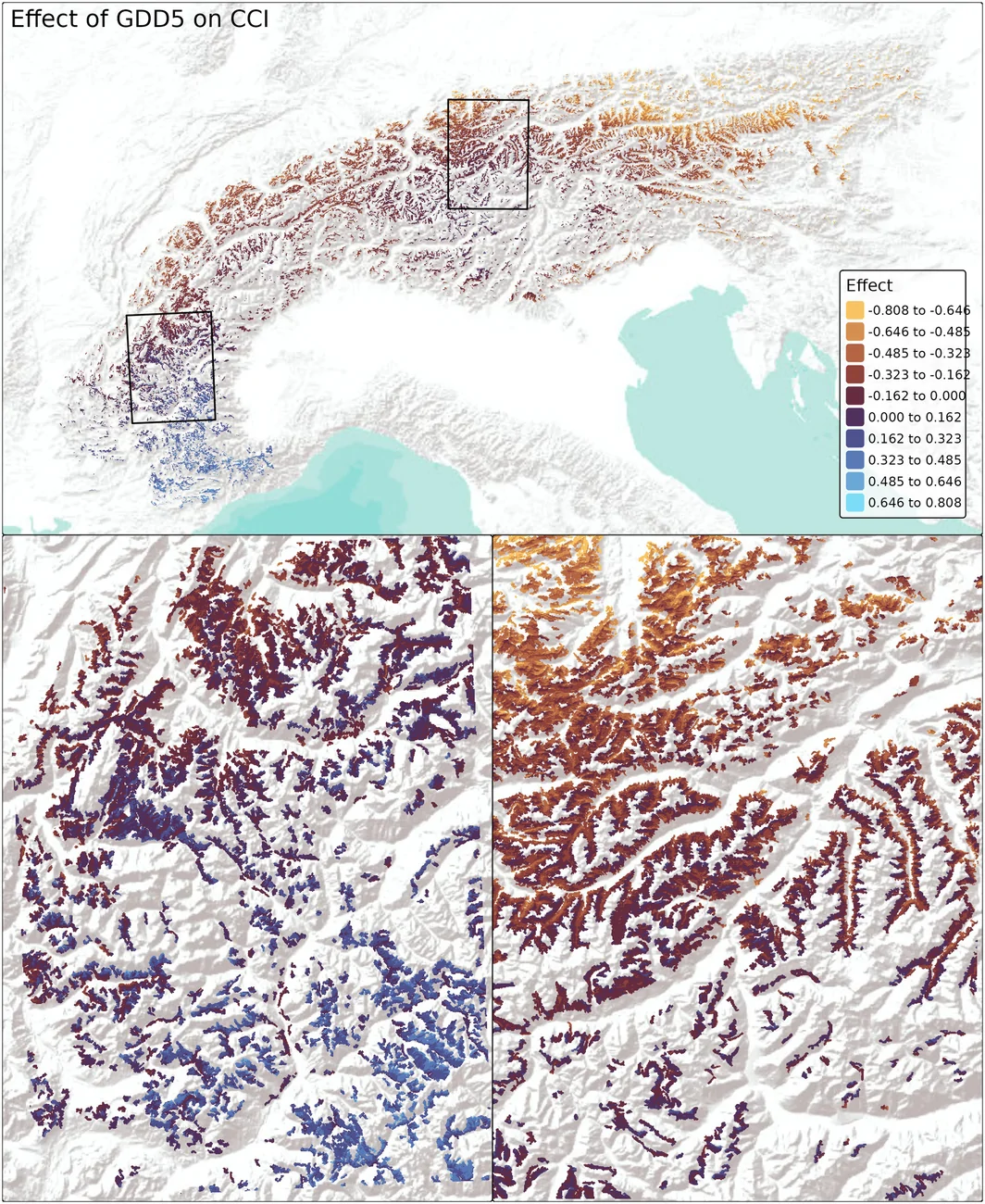
Predicted spatial distribution of the within‐plot GDD → CCI effect from the varying‐coefficient LMM. Colors show the magnitude and direction of the GDD → CCI effect on the asinh scale (blue = more negative; orange = more positive).

#### 3.2.2 Focus on GDD → NIRv Environmental Modulation

The fitted GDD → NIRv relationship showed substantial spatial heterogeneity, with static environmental variables accounting for approximately 30% of plot‐to‐plot variation in effect size. Spatial‐bootstrap inference supported three climatic moderators (Figure 5): the positive GDD → NIRv response strengthened with higher mean annual precipitation and greater temperature seasonality, but weakened with greater baseline winter snow accumulation. Moderator estimates for mean elevation, long‐term CWD, soil pH, soil sand content, and heat load had 95% bootstrap intervals overlapping zero and are therefore not interpreted as supported effects. The spatial projection in Figure 7 shows the geographic pattern predicted by the full varying‐coefficient model, with generally stronger positive responses in wetter regions and weaker responses in snow‐rich areas. As for Figure 6, finer‐scale spatial contrasts should be considered descriptive because the map combines supported and uncertain moderator coefficients.

**FIGURE 7:**
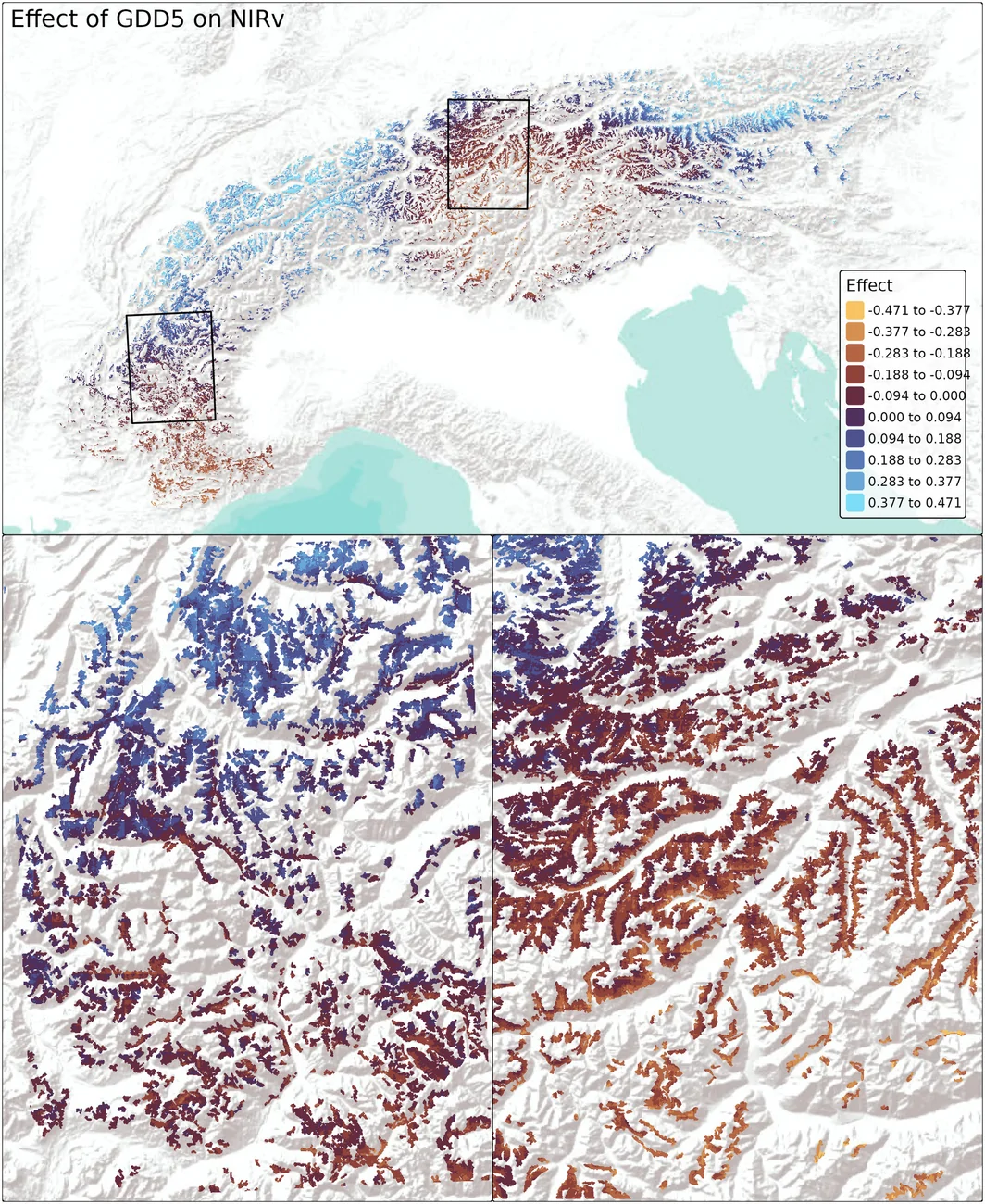
Predicted spatial distribution of the within‐plot GDD → NIRv effect from the varying‐coefficient LMM. Colors show the magnitude and direction of the GDD → NIRv effect on the *asinh* scale (blue = more negative; orange = more positive).

Complete moderator results for all focal pathways and additional spatial projections are provided in Supporting Information Section S7.

## 4 Discussion

By combining high‐resolution remote sensing with panel data and mediation models, we found that interannual variation in alpine grassland productivity was consistent with concurrent direct climatic pathways and indirect pathways through integrated canopy functional properties, the strengths of which varied with environmental context.

### 4.1 Direct and Indirect Climatic Effects on Productivity

Warmer growing seasons were associated with lower CCI, indicating a lower canopy chlorophyll‐to‐carotenoid pigment balance and relatively greater carotenoid investment during warmer years. This pattern is consistent with increased photoprotective demand and physiological stress during warmer years, consistent with enhanced evaporative demand and soil moisture depletion (Vicca et al. 2016; Yuan et al. 2019).

However, after accounting for residual spatial dependence, the GDD effects on CIre and NDII were uncertain, so the evidence does not support a general decline across all pigment‐ and water‐related canopy properties. Similarly, the positive SWE–NDII association was not supported by the spatial‐bootstrap interval. Climatic water deficit (CWD) reduced canopy water content (NDII) and within‐plot spectral functional heterogeneity (FRic), consistent with drier years constraining canopy water status and narrowing the range of pixel‐integrated canopy states expressed within plots. This reduction in spectral heterogeneity is consistent with expectations of the environmental filtering theory, with increased stress reducing the variation of functional strategies expressed (Cornwell et al. 2006; Le Bagousse‐Pinguet et al. 2017). This is also consistent with the trait driver theory, which predicts that shifts in trait means and dominance are expected to precede changes in trait diversity (Enquist et al. 2015). However, the absence of supported effects of GDD and SWE on FRic indicates that short‐term diversity responses were specific to climatic drivers.

Interannual variation in pigment‐related canopy properties was positively associated with productivity. This relationship is consistent with mass‐ratio expectations, whereby dominant physiological strategies control ecosystem functioning (Enquist et al. 2015; Grime 1998). Higher canopy chlorophyll content and a higher chlorophyll‐to‐carotenoid pigment balance were consistently associated with higher productivity. The positive relationships between pigment‐related spectral properties and NIRv may reflect the combined influence of pigment investment, vegetation amount and canopy structure on absorbed radiation and carbon uptake. These components are biologically coupled at the ecosystem level, but their individual contributions cannot be separated fully using the present multispectral observations. Consequently, the estimated indirect pathways represent integrated changes in realized canopy functioning and should not be interpreted as LAI‐independent effects of leaf physiology. Contrary to our expectations, we found a negative, although weak, effect of canopy water status on productivity, which may reflect energy–water co‐limitation under cooler or cloudier conditions (Damm et al. 2018). Within‐plot spectral functional heterogeneity was negatively associated with vegetation productivity, indicating that years with a broader range of pixel‐integrated canopy states across sub‐plot locations tended to be less productive. This relationship should not be interpreted as definitive evidence of a directional effect, because both patterns may arise jointly during unfavorable years. At the spatial and temporal resolution of our observations, greater within‐plot spectral heterogeneity may arise from several non‐exclusive processes, including spatial variation in species or functional‐group dominance, physiological plasticity, phenological asynchrony, canopy structure and fine‐scale environmental heterogeneity. Climatic stress or phenological asynchrony could, for example, increase spatial variation in canopy states within a plot while simultaneously reducing seasonally integrated productivity. Thus, this result should not be interpreted as evidence that greater individual‐ or species‐level alpha functional diversity reduces productivity. Rather, it indicates that years with greater spatial heterogeneity in community‐integrated canopy states tended to have lower productivity. Interannual fluctuations in FRic are therefore likely to reflect changing spatial patterns of physiological state, phenology and relative dominance within plots, rather than species turnover alone. Over longer community assembly trajectories, however, complementarity and insurance effects of ecological biodiversity may still enhance productivity, stability, and resilience, as demonstrated in classical biodiversity–ecosystem functioning experiments (Isbell et al. 2015; Loreau and de Mazancourt 2013; Tilman et al. 2014); these mechanisms are not directly measured by the FRic‐based spatial heterogeneity metric used here.

The estimated direct pathway from growing‐season thermal accumulation to annual productivity was strongly positive, while greater snow accumulation had a negative effect. These results directly support our prediction that ecosystem productivity in mountain grasslands is primarily constrained by cumulative energy availability and growing‐season length, thus being independent of short‐term trait reorganization. In such energy‐limited systems, higher thermal accumulation has been found to enhance productivity by extending the seasonal period suitable for carbon assimilation and high metabolic rates (Piao et al. 2011). In addition, greater snow accumulation tends to delay snowmelt, leading to a shorter effective growing season and thus lower annual productivity (Körner 2021b; Wipf and Rixen 2010).

Our structural equation models revealed that these processes might operate through two concurrent pathways: a direct energy‐driven pathway and an indirect pathway mediated by canopy functional structure. This general finding of partitioned direct and indirect effects mirrors our expectation, according to which climatic controls on ecosystem functioning operate alongside, and sometimes in opposition to, trait‐mediated pathways.

For temperature accumulation (GDD), the negative indirect effect via a lower chlorophyll‐to‐carotenoid pigment balance (CCI) contradicted, but did not outweigh, the positive direct effects of longer and warmer growing seasons, ultimately resulting in higher net productivity. Thus, warmer years were associated with greater productivity despite a concurrent shift in canopy pigment balance. The coexistence of these opposing mechanisms could explain why we found increased productivity during warmer years despite simultaneous evidence of physiological stress within the canopy. Together, this may indicate that primary productivity is governed by constraints on the cumulative amount of energy available across the year, rather than by physiological limitations alone (Del Grosso et al. 2008; Fatichi et al. 2019; Nemani et al. 2003; Stephenson 1998). For SWE, the negative direct effect dominated, yielding a negative total effect, while none of the canopy‐mediated pathways were supported. The negative effects of climatic water deficit (CWD) on NDII and FRic were individually supported and, because both canopy properties were negatively associated with productivity, generated positive trait‐mediated effects. However, the direct effect of CWD on productivity remained uncertain and was opposite in sign. These direct and trait‐mediated pathways therefore appeared to offset one another, resulting in summed indirect and total effects that were not distinguishable from zero.

Overall, this decoupling between physiological condition and productivity suggests that short‐term productivity gains under warmer conditions may thus come at the cost of reduced canopy functional integrity, potentially constraining the persistence of these gains under continued climate change (De Bello 2021; Dee et al. 2023).

### 4.2 Environmental Modulation of Estimated Pathways

A key result of this study was the finding that direct and indirect climatic effects on vegetation were context‐dependent. While the context dependence of climate–vegetation relationships is well established (Corona‐Lozada et al. 2019; Denissen et al. 2022; Seddon et al. 2016), our contribution lies in quantifying how environmental context alters the balance between direct climatic pathways and indirect pathways through canopy functional properties. Consistent with previous work showing that vegetation sensitivity to climate varies with background moisture, climatic regime and topographic setting (Corona‐Lozada et al. 2019; Hoylman et al. 2019; Seddon et al. 2016), the strength and direction of these relationships varied substantially across environmental gradients of climate, soil, and topography. Across pathways, climatic moderators generally showed the broadest supported modulation, while topographic conditions modified selected relationships and supported soil effects were generally smaller. This pattern is consistent with macroclimate providing an important background constraint on short‐term vegetation responses across the Alps, without implying that topography is uniformly secondary (Dee et al. 2023; van der Plas 2019). In contrast, however, we found that the effects of canopy functional structure on ecosystem functioning were comparatively weakly modulated by environmental context. This indicates that canopy‐property–productivity relationships showed limited short‐term modulation by environment, suggesting that dominant trait mechanisms may override context‐dependent complementarity effects at interannual timescales (Isbell et al. 2015; Loreau 1998; van der Plas 2019).

In the H1 pathways (climate → canopy properties), spatial‐bootstrap support for environmental moderation of the GDD → CCI relationship was strongest for mean annual temperature, mean elevation, and temperature seasonality. The negative GDD → CCI association was weaker in warmer baseline climates and at higher elevations, but stronger where temperature seasonality was greater. Other apparent spatial modifiers, including long‐term climatic water deficit and mean annual precipitation, did not show robust moderator effects. These results highlight the joint role of baseline climate and topography in shaping canopy pigment responses to interannual warming, while indicating that the most robust modifiers were thermal context and elevation. The spatial projection in Figure 6 is therefore used to illustrate the fitted geographic pattern rather than to assign statistical support to every local contrast.

In the H2 pathways (canopy properties → functioning), environmental modulation was weak overall. Integrated canopy‐property–productivity relationships were relatively stable across background climatic, soil, and topographic gradients.

In the H3 pathways (climate → functioning), spatial‐bootstrap inference indicated that the positive effect of growing‐season thermal accumulation on productivity strengthened with higher long‐term precipitation and greater temperature seasonality, but weakened with greater baseline winter snow accumulation. The precipitation pattern suggests that greater water availability allows alpine grasslands to benefit more from warm years, whereas water limitation constrains this productivity response in drier areas (Choler 2023; Corona‐Lozada et al. 2019).

Together, our results highlight a coherent picture in which the environment exerts hierarchical control of productivity in both time and space. Specifically, spatial variation in (macro)climate and local topography modulates both plant physiological responses and ecosystem productivity responses to interannual variability in climate. Together, these results underscore the importance of “response diversity” at regional scales, whereby different environmental contexts and functional composition provide alternative pathways for ecosystem function–supporting responses to climatic forcing (Elmqvist et al. 2003; Seddon et al. 2016).

### 4.3 Perspectives and Limits

Our findings highlight several directions for future research. First, the strong spatial variability in estimated pathway directions cautions against universal expectations of biodiversity–ecosystem functioning relationships, as functional composition and diversity can buffer, amplify, or decouple climate impacts depending on the environmental context. The direct energy‐ and water‐limitation pathways may generalize to other temperate mountain grasslands, but their magnitudes and the signs of indirect canopy pathways are likely to depend on regional snow regimes, aridity, land use, and community composition. Replication of this or similar study frameworks in other mountain systems is therefore necessary for understanding other similar mountainous biomes. Second, extending analyses beyond productivity to ecosystem stability, resilience, and multifunctionality will help evaluate the long‐term consequences of biodiversity change (De Bello 2021; Isbell et al. 2015). Third, the comparatively weaker and spatially stable canopy‐property–productivity relationships that we observed likely reflect the short temporal scope of our study and the omission of delayed community and functional responses to climate. Incorporating lagged community and trait dynamics is therefore a key priority for further enhancing our understanding of long‐term ecosystem resilience (Alexander et al. 2015, 2018; Dullinger et al. 2012). Fourth, climatic effects on productivity are likely non‐linear, highlighting the need to explicitly consider thresholds and saturation effects under continued warming (Körner 2021b). Remote‐sensing uncertainty also remains incompletely propagated: residual atmospheric effects, view geometry, mixed pixels, and unequal numbers of clear observations may contribute to year‐to‐year variation despite quality masking, the vegetation‐cover filter and percentile compositing. Our intervals therefore quantify model and spatial‐sampling uncertainty conditional on the processed remote‐sensing variables, not total observation error. Finally, linking spectral proxies more directly to species‐level trait databases, integrating belowground processes, and accounting for land‐use and management drivers will be essential for developing comprehensive, policy‐relevant maps of ecosystem responses to global change.

### 4.4 Conclusion

By integrating remote sensing with panel‐data and mediation models, our study provides a spatially explicit framework for separating direct climatic pathways from indirect pathways through canopy functional properties. The results identify where short‐term productivity responses to warming are associated with greater energy availability and where canopy or water constraints may limit those responses. These conditional estimates should be tested across longer time series and other mountain systems before being generalized to long‐term ecosystem trajectories.

## Author Contributions

**Gaüzère Pierre:** conceptualization, writing – original draft, methodology, validation, visualization, writing – review and editing, formal analysis, data curation. **Maria Hällfors:** funding acquisition, writing – review and editing. **Jussi Mäkinen:** writing – review and editing. **Philippe Choler:** writing – review and editing. **Risto K. Heikkinen:** writing – review and editing. **Romain Goury:** writing – review and editing, conceptualization. **Joaquim Estopinan:** conceptualization, writing – review and editing. **Cyrille Violle:** writing – review and editing. **Franziska Schrodt:** writing – review and editing. **Sara Si Moussi:** conceptualization, writing – review and editing. **Mickaël Hedde:** writing – review and editing. **Luca Santini:** writing – review and editing. **Wilfried Thuiller:** conceptualization, writing – review and editing, funding acquisition.

## Funding

This work was supported by the HORIZON EUROPE Framework Programme (101134954) and the Fondation pour la Recherche sur la Biodiversite (IMPACTS).

## Conflicts of Interest

The authors declare no conflicts of interest.

## Supporting information


**Data S1:** Text S1 Mechanistic expectations underlying hypotheses H1–H4.
**Supporting Information: Text S2** Pair plots of vegetation indices.
**Supporting Information: Text S3** Extended Methods.
**Figure S4:** Comparison of within‐plot coefficient estimates obtained with the demeaned linear mixed‐model (LMM) and two‐way fixed‐effects (TWFE) approaches. Panels correspond to H1 (climate → canopy properties), H2 (canopy properties → productivity, NIRv), and H3 (climate + canopy properties → NIRv). Salmon circles show LMM estimates and teal triangles show TWFE estimates; horizontal intervals indicate the corresponding 95% confidence intervals. Both approaches use the same variables, transformations, controls and year effects and target the same within‐plot estimand. The close agreement in effect magnitude and direction indicates that the main conclusions are robust to the choice of modeling framework.
**Supporting Information: Text S5** Decomposition of climate effects into direct, indirect, and total components.
**Supporting Information: Text S6** Indirect climate effects, decomposed by mediator.
**Supporting Information: Text S7** Environmental controls and mapping.
**Figure S5:** Direct, summed indirect, and total associations of interannual climate anomalies with vegetation productivity. Black points show the effect estimates obtained from the complete dataset. Blue horizontal intervals show the 2.5th–97.5th percentile intervals obtained from 1,000 common‐resample 150‐km spatial‐block bootstrap replicates. Direct effects correspond to the conditional climate coefficients in the productivity model, summed indirect effects are the sum of the mediator‐specific climate → canopy property → NIRv path products, and total effects are the sum of the direct and summed indirect components. Intervals overlapping zero indicate effects that were not statistically supported under the spatial‐bootstrap inference.
**Figure S6:** Mediator‐specific indirect associations of interannual climate anomalies with vegetation productivity. Black points show the mediator‐specific indirect effect estimates obtained from the complete dataset for each climate → canopy property → NIRv pathway. Blue horizontal intervals show the 2.5th–97.5th percentile intervals obtained from 1,000 common‐resample 150‐km spatial‐block bootstrap replicates. These indirect effects correspond to the products of the climate → canopy‐property and canopy‐property → NIRv path coefficients for each mediator separately. For FRic, the mediator denotes within‐plot spectral functional heterogeneity among pixel‐integrated canopy states. Intervals overlapping zero indicate mediator‐specific indirect effects that were not statistically supported under the spatial‐bootstrap inference.

## Data Availability

The data and code supporting the findings of this study are openly available in Zenodo at https://doi.org/10.5281/zenodo.18670711. The repository contains the data‐processing and analysis scripts and associated reproducibility materials required to reproduce the analytical workflow, statistical analyses, and figures presented in the article. The original remote‐sensing, climate, and environmental data used to construct the analytical dataset are derived from publicly available sources, including Sentinel‐2, ERA5‐Land, TerraClimate, WorldClim, Copernicus DEM, OpenLandMap, and HydroATLAS; details of the source datasets and processing workflow are provided in the repository and in the Supporting Information.
