# Supplementary material for: Direct and Trait‐Mediated Effects of Climate on Grassland Productivity Across the Alps
Source: Glob Chang Biol. 2026 Oct 16;32(10):e71142. doi: 10.1111/gcb.71142 (PMC13675600; doi:10.1111/gcb.71142)
Supplement: Supplementary file 1 [file GCB-32-e71142-s001.pdf]

Supporting Information

S1 — Mechanistic expectations underlying hypotheses H1–H4

H1 — Climate → canopy functional composition and within-plot spectral functional heterogeneity (FRic).
Growing-season thermal accumulation (GDD) increase chlorophyll investment (↑CIre) and reduce photoprotective demand (↑CCI) because more favorable growing seasons relaxes thermal constraints, allowing plants to invest more in photosynthetic pigments and reduce relative photoprotective pigment ratios. Alternatively, if high GDD coincides with water limitation and heat stress, it can decrease chlorophyll (↓CIre) and increase photoprotective demand (↓CCI). Higher growing-season water deficits (CWD) lower leaf water content and pigment maintenance (↓NDII, ↓CIre; ↓CCI via higher carotenoid fractions).
More snow during the winter (SWE) leading to late snowmelt reduces the window for chlorophyll pigment gain (↓CIre) but decreases frost risk (Körner, 2021b; Wipf & Rixen, 2010). Alternatively, if water limitation dominates, extra heat and less snow reduce the amount of pigments and increase water deficit (↓CIre, ↓CCI, ↓NDII) despite longer growing seasons. From a Trait Driver Theory perspective, these expectations arise because climatic anomalies act as environmental filters that shift the distribution of expressed physiological traits at the community level, either through changes in dominance or within-species physiological adjustment (Enquist et al., 2015; Violle et al., 2007)**.**
Within-plot spectral functional heterogeneity (FRic) may increase if climatic variability generates divergent pixel-integrated canopy states across sub-plot locations, and may decrease if climatic stress homogenizes those states. Alternatively, FRic may remain stable if climatic anomalies affect canopy states similarly across the plot or if spatial reorganization is limited over annual timescales. Because each Sentinel-2 pixel integrates the canopy signal of multiple individuals and potentially multiple species, FRic here describes spatial heterogeneity among community-integrated canopy states rather than individual- or species-level alpha functional diversity and is conceptually closer to sub-plot beta functional diversity (Rossi et al., 2020; Zhao et al., 2025).

H2 — Canopy functional properties → productivity.
CIre/CCI/NDII primarily reflect mass-ratio control via dominant canopy strategies, whereas FRic quantifies within-plot spectral functional heterogeneity among pixel-integrated canopy states. Higher chlorophyll-related reflectance (↑CIre) and lower photoprotective demand (↑CCI) increases seasonal productivity (↑NIRv), while higher canopy water content (↑NDII) supports higher productivity when energy supply is sufficient (Grime, 1998; Loreau & Hector, 2001). Trait Driver Theory explicitly predicts that ecosystem functioning should scale with the abundance-weighted distribution of functional traits expressed in the canopy, rather than with species or trait richness per se (Enquist et al., 2007, 2015; Shipley et al., 2016). Thus, strong links between pigment- and water-related spectral traits and productivity are expected. Greater within-plot spectral functional heterogeneity (FRic) may be associated with productivity, but its sign is not fixed a priori because spatial heterogeneity can arise from differences in dominance, physiological state, phenology, canopy structure, or fine-scale environmental conditions. At interannual scales, FRic–productivity associations could be weak or negative if greater spatial heterogeneity reflects stress responses that dilute the dominance of canopy states that efficiently convert absorbed light into carbon (Loreau & de Mazancourt, 2013). In wet or cool years NDII could rise while NIRv falls if radiation limits photosynthesis. Under Trait Driver Theory, a weak or negative FRic–productivity relationship can occur if greater within-plot spectral heterogeneity accompanies a relaxation of dominance by highly productive canopy states; however, FRic itself should not be interpreted as individual- or species-level alpha functional diversity (Enquist et al., 2015).

H3 — Climate → Functioning.
Expectation: higher GDD will directly increase productivity reflecting the extended and warmer growing season (↑NIRv), whereas higher growing-season water deficit (CWD) should decrease productivity (↓NIRv).

Higher snow water equivalent (SWE) can decrease productivity when decreasing season length (later snowmelt) is not offset by frost or higher evaporative demand (Körner, 2021a; Wipf & Rixen, 2010). Alternatively, in warm years with drought may result in muted or neutral net effects in functioning if water stress cancels out the benefits of energy gains. These direct effects correspond to climatic controls acting on ecosystem-level fluxes independently of community trait reorganization, reflecting constraints on energy input and carbon assimilation rather than shifts in trait distributions

H4 — Environmental control and spatial prediction.

Background environmental conditions modulate the effects of H1-3. Our main expectation in H1 (climate→canopy-property relationships), is that the response of spectral traits to climate anomalies should be modulated across large scales based on climatic baseline conditions. Subsequently, at smaller spatial scales, topography and soil are also expected to modulate the response, as insolation and soil water retention capacity can intensify or mitigate pigment content and water stress. Trait Driver Theory predicts that the sensitivity of trait distributions to climatic anomalies should vary systematically along environmental gradients, as baseline conditions determine both the pool of viable strategies and the strength of environmental filtering. Our main expectation for H2 (canopy-property→productivity relationships), is that positive links exist between chlorophyll-related composition (CIre, higher CCI) and NIRv, and that these are strongest in high moisture areas with high insolation. Thus, FRic–productivity associations could weaken under more stressful environmental conditions (coldest or driest) if spatial heterogeneity among canopy states is reduced, but could also change sign where harsher environments generate stronger spatial differentiation among canopy states. Our main expectation for H3 (climate→productivity effects), is that in wetter and lower insolation contexts warming benefits on NIRv should be amplified, whereas high elevation, cold, snow-dominated, or highly insolated sites with coarse/alkaline soils should shift responses toward stress-limited regimes and thus attenuate warming benefits.

S2 - Pair plots of vegetation indices

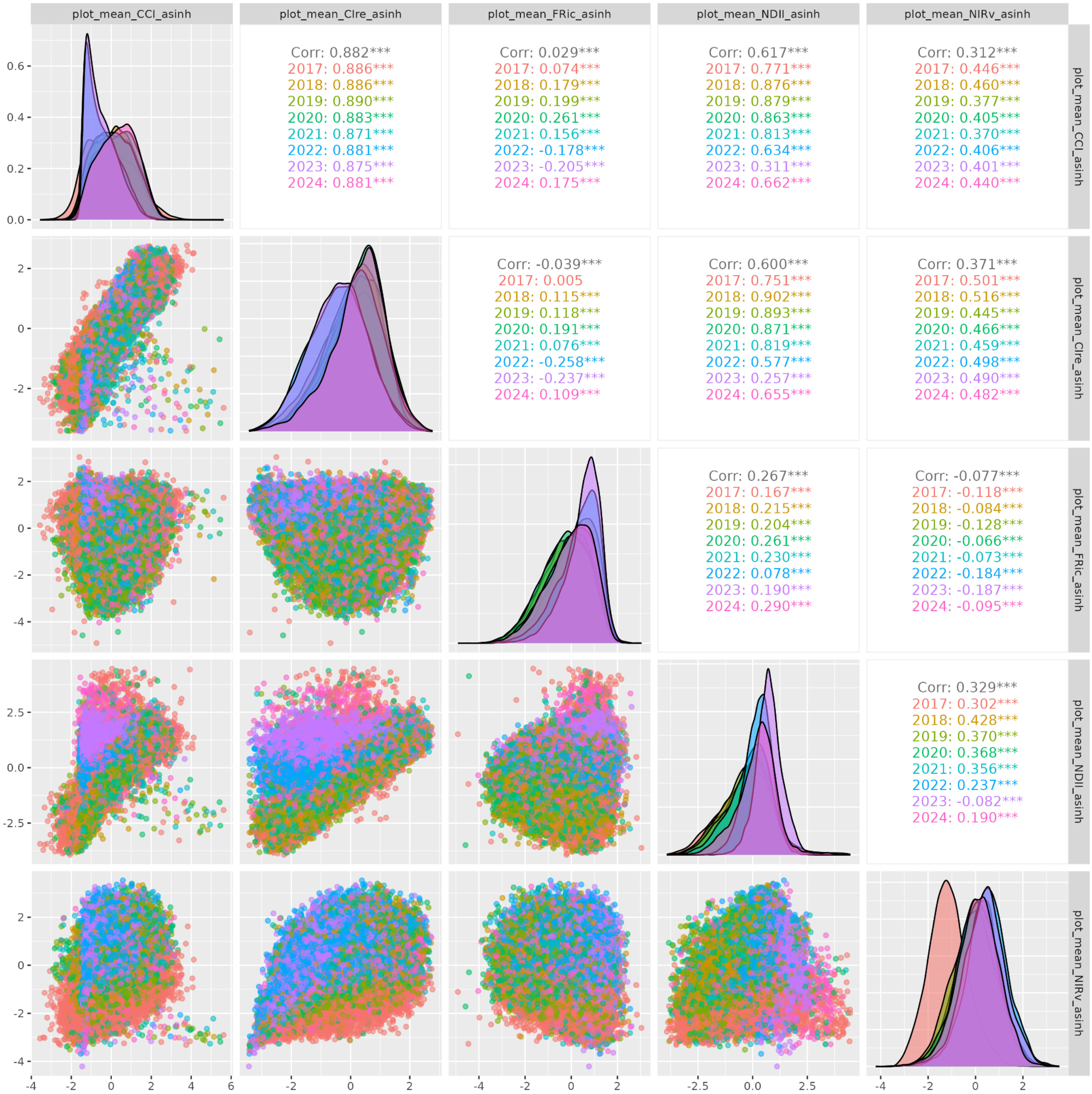

The observed covariance among spectral indices and productivity is consistent with climate acting on integrated canopy properties (leaf area, pigment investment, structure) that simultaneously regulate light absorption and carbon uptake, rather than on independent trait dimensions

S3 - Extended Methods

This section provides the full technical description of data sources, preprocessing workflows, statistical models, and robustness analyses underlying the main-text Methods, which are presented in a condensed form for clarity. Note that all the scripts to generate the data are available directly on Google earth engine, see data availability for the links.

Data and processing

Remotely sensed vegetation time series

We used Sentinel-2 Level-2A surface reflectance imagery (10–20 m spatial resolution) from the COPERNICUS/S2_SR_HARMONIZED collection. All processing was conducted in Google Earth Engine. For each year between 2017 and 2024, imagery was restricted to the growing season (April–October). Cloud and cirrus contamination were masked using QA60 bits 10 and 11, and snow and ice were removed using the Scene Classification Layer (SCL = 11).

To ensure reliable estimation of spectral indices in alpine environments, pixels with low vegetation signal were excluded prior to index computation. Specifically, pixels with fractional vegetation cover below 0.6 were filtered out, thereby minimizing contamination from bare ground, rocks, senescent litter, or mixed surfaces known to bias pigment- and water-related indices. FVC was calculated for each Sentinel-2 observation from NDVI using a linear vegetation–soil mixture model:

$$FVC = \mathrm{clamp}[(NDVI - NDVIsoil) / (NDVIveg - NDVIsoil), 0, 1]$$

where NDVIsoil = 0.20 represents the bare-soil limit and NDVIveg = 0.86 represents the dense-canopy limit. We retained pixels with FVC ≥ 0.60, corresponding to NDVI ≥ 0.596 under this model.

The value of 0.60 was selected as a compromise between retaining the relatively open structure of mountain grasslands and ensuring that vegetation remained the dominant component of the pixel signal. It does not require a closed or dense canopy, but ensures that the estimated vegetation fraction is at least 60%. Below this threshold, a substantial proportion of the reflectance may originate from soil, rock, litter or other non-vegetated surfaces. Changes in the resulting spectral indices may then reflect variation in the relative contributions of vegetation and background surfaces rather than variation in canopy pigment, water status or productivity.

Remote-sensing measurement uncertainty. The quality masks, fractional-vegetation-cover filter and percentile compositing reduce contamination, but they do not eliminate residual atmospheric effects, bidirectional-reflectance and view-angle variation, geolocation error, mixed pixels, or unequal numbers and timing of cloud-free observations among plots and years. These sources of observation error were not propagated explicitly through the statistical models. Consequently, the reported spatial-bootstrap intervals quantify uncertainty arising from spatial dependence and model estimation conditional on the processed indices, rather than the total uncertainty of the original satellite observations.

Surface reflectance bands (B1–B12) were scaled to unitless reflectance and constrained to the [0, 1] interval.

All retained pixels within each 60 m-radius plot were processed independently prior to spatial aggregation.

Within-plot spectral functional heterogeneity (FRic). To quantify spatial heterogeneity in canopy functional states, we applied the functional richness (FRic) metric to the multivariate spectral trait values of all retained Sentinel-2 pixels within each plot-year. FRic measures the extent of the spectral trait space

occupied by pixel-integrated canopy states within a plot. Because each Sentinel-2 pixel integrates the canopy signal of multiple individuals and potentially multiple species, variation among pixels is not interpreted as individual- or species-level alpha functional diversity. Instead, FRic captures spatial heterogeneity among community-integrated canopy states at sub-plot scales and is conceptually closer to beta functional diversity (Rossi et al., 2020; Zhao et al., 2025).

Spectral traits as proxies of realised canopy functional structure. We used three spectral indices to characterize canopy functional structure: a red-edge chlorophyll index (CIre), a chlorophyll–carotenoid index (CCI), and a near-infrared water index (NDII). These indices reflect complementary aspects of plant physiological functioning, including pigment investment, photoprotective demand, and canopy water status. The estimated spectral traits function as proxies for three key leaf-level physiological traits that capture complementary aspects of vegetation functioning: photosynthetic capacity, photoprotection, and leaf water status (Gitelson et al., 2006). More precisely, CIre is a proxy for leaf chlorophyll content, with higher CIre values indicating greater chlorophyll content and higher potential photosynthetic activity (Ali et al., 2020; Croft et al., 2017); CCI is sensitive to the balance between chlorophyll and carotenoid pigments; lower CCI values indicate a lower chlorophyll-to-carotenoid balance and relatively greater carotenoid investment, consistent with increased photoprotective demand and reduced photosynthetic activity (Gamon et al., 2016); NDII is a proxy for leaf water content, and reflects plant water status and strategies of water use and its conservation. Although indirect, this set of spectral traits have the advantage of being ecologically complementary (Schneider et al., 2017), providing independent information about photosynthetic, photoprotective, and water-use strategies (Damm et al., 2018). Rather than interpreting them as direct measurements of species composition, we treat these indices as proxies of realised canopy functional structure, integrating both changes in species dominance and within-species physiological adjustment to interannual climatic variability. Because these indices capture expressed canopy properties, variation in CIre, CCI, and NDII can arise from multiple processes operating simultaneously, including shifts in species relative abundance (mass-ratio effects) and phenotypic or physiological plasticity within species. Consequently, indirect pathways estimated in our mediation framework should be interpreted as operating through integrated canopy functional properties, rather than as evidence of taxonomic turnover.
Three spectral indices were derived to characterize canopy pigment investment and water status:

- **Red-Edge Chlorophyll Index (CIre)**

  $$CIre = \frac{B7}{B5} - 1$$

  Following (Clevers & Gitelson, 2013), using Sentinel-2 red-edge bands B5 and B7.

- **Chlorophyll-Carotenoid Index (CCI)**

  $$CCI = \frac{B3 - B4}{B3 + B4}$$

  following (Gamon et al., 2016) and (Helfenstein et al., 2022), based on visible bands B3 and B4. This Sentinel-2 implementation is sensitive to the relative chlorophyll-to-carotenoid pigment balance rather than absolute carotenoid content.

- **Normalized Difference Infrared Index (NDII)**

  $$NDII = \frac{B8A - B11}{B8A + B11}$$

  following (Hardisky et al., 1983), using near-infrared (B8A) and short-wave infrared (B11) bands.

To summarize the seasonal physiological state while reducing residual noise from clouds, view angle effects, or short-term phenological fluctuations, CIre, CCI, and NDII were composited using the 80th percentile of all

valid observations within each growing season and plot. This percentile-based compositing targets near-peak physiological conditions while avoiding extreme outliers.

CIre, CCI, and NDII quantify canopy-level pigment investment and water status derived from reflected radiation and are therefore treated as indicators of realised canopy functional structure rather than as direct measurements of species composition. Variation in these indices may arise from changes in species relative abundance or identity as well as from within-species physiological or phenotypic adjustment to interannual climatic conditions. Because canopy biophysical properties (e.g. leaf area, pigment pools, and structure) jointly influence multiple spectral bands, CIre, CCI, NDII, and productivity metrics are expected to covary at the plot scale. **In the mediation framework used here, this shared variance is treated as a meaningful signal associated with integrated canopy functioning rather than as a redundant radiometric artefact** (see Figure S2 for pairwise relationships among indices).

Vegetation productivity

Vegetation productivity was quantified using near-infrared reflectance of vegetation (NIRv), following (Badgley et al., 2017):

$$NIRv = \left(\frac{B8 - B4}{B8 + B4}\right) \times B8$$

where B8 and B4 correspond to Sentinel-2 near-infrared and red bands, respectively. NIRv provides a linear proxy of gross primary productivity across a wide range of ecosystems and is particularly robust when integrated over time.

To estimate cumulative growing-season productivity, NIRv was computed for each valid Sentinel-2 acquisition between April and October and summed across the season for each pixel. All 10 m pixels within each 60 m-radius plot were retained, and pixel-level values were exported for each plot–year combination. This procedure leverages the high revisit frequency of Sentinel-2 (≈5 days) to approximate seasonal integration of canopy carbon uptake while minimizing contamination from clouds and snow. **NIRv** provides a linear proxy for gross primary production (GPP) in most ecosystems, and is particularly reliable when integrated over the growing season (Yu et al., 2024; Zheng et al., 2024). The resulting dataset provides, for each plot and year, (i) near-peak estimates of canopy pigment and water-status traits and (ii) an integrated metric of growing-season productivity suitable for subsequent plot-level causal analyses.

Interpretation of spectral mediators. The indices CIre, CCI, and NDII reflect canopy-level pigment investment and water status, and are therefore treated here as metrics of realised canopy functional structure rather than direct measurements of taxonomic or trait composition. Variation in these indices can arise from (i) changes in species' relative abundances and identities (mass-ratio–type shifts), and/or (ii) within-species phenotypic and physiological adjustment to interannual conditions. Consequently, indirect pathways estimated in our piecewise SEM should be interpreted as operating through integrated canopy functional properties, not as evidence of compositional turnover. Because canopy biophysical properties jointly influence multiple spectral bands, these indices are expected to covary at a plot scale (Figure S2). In our mediation framework, this shared variance is treated as a signal of integrated canopy functioning rather than as redundant radiometric noise.

Climate interannual variability

We characterized interannual climatic variability using three variables selected to represent the primary abiotic constraints on mountain grassland functioning: thermal energy availability, water limitation, and snow dynamics. These variables were chosen based on well-established theoretical and empirical links between climate, plant physiological activity, and growing-season length in mountain ecosystems (Körner, 2021b; Stephenson, 1998; Wipf & Rixen, 2010).

Thermal conditions were quantified using growing degree days above 5 °C (GDD), which integrates both temperature magnitude and growing-season duration and is a standard proxy for thermal energy available for plant growth. Water limitation was characterized using growing-season climatic water deficit (CWD), which captures the balance between atmospheric demand and water supply and has been shown to be a key determinant of plant stress and productivity. Snow dynamics were represented by maximum winter snow water equivalent (SWE), reflecting the role of snowpack in regulating soil moisture recharge, frost protection, and the timing of snowmelt and growing-season onset.

Climate time series were extracted for each plot for the period 2017–2024 from gridded reanalysis and climatological datasets (ERA5-Land hourly reanalysis at 9km resolution for GDD, TerraClimate monthly fields at ~4km for CWD and SWE). Although the native spatial resolution of these products is coarser than the plot scale, our inference targets within-plot interannual anomalies rather than fine-scale spatial gradients. Consequently, climate variables are interpreted as year-to-year deviations experienced by each plot relative to its own mean climatic context, rather than as precise microclimatic estimates. This approach is appropriate for isolating temporal climatic forcing while controlling for persistent spatial differences among sites.

Static environmental variables

To characterize the background environmental context in which interannual climate variability operates, we compiled a set of **static environmental variables** describing long-term climatic conditions, soil properties, and topography across the entire study area. We retained a theory-driven, low-collinearity set of variables for analyses including climatic, soil, and topographic descriptors. The static climatic descriptors that were retained were Climatic Water Deficit (CWD, mm), Mean Annual Precipitation (MAP, mm), Mean Annual Temperature (MAT, °C, lapse rate downscaled at 100m), SWE (mm), and Temperature seasonality extracted from WorldClim BIO layers (BIO1 for annual mean temperature, BIO4 for temperature seasonality, BIO12 for annual precipitation), and TerraClimate monthly fields to obtain the mean annual climatic water deficit (CWD) over 2001–2020, and the snow water equivalent baseline as the mean, across years, of the hydrologic-winter (October of year y–1 to May of year y) maximum of monthly snow water equivalent. The static soil descriptors that we retained were lithology, pH, sand percentage, and soil organic content. Lithology was assessed by rasterizing the HydroATLAS (GLiM-derived) majority lithology class to 100 m. pH,

sand percentage, and soil organic content were obtained from OpenLandMap (v02/v01) at 250 m resolution and depth-weighted to 0–30 cm (0–10 and 10–30 cm layers). The topographic predictors that we retained were Heat Load Index (HLI), mean elevation, and elevation range, computed from Copernicus Digital Elevation Model at 30 m resolution (Copernicus GLO-30). Mean elevation and elevation range were both aggregated from 30 m to 100 m resolution. HLI was computed by McCune–Keon formulation using slope, aspect (at 30 m resolution), and latitude, then aggregated at 100 m resolution. All static variables were harmonized on a continuous 100 m grid (EPSG:3035) covering the grassland mask (study area) and processed with consistent projections to avoid projection artifacts.

Analyses

Study design and variables.
We quantified within-year relationships among climate anomalies, realised canopy functional properties and ecosystem productivity across 10,000 Alpine grassland plots. For H1 (climate → canopy properties), the outcomes were CIre, CCI, NDII and **within-plot spectral functional heterogeneity (FRic), and the predictors were GDD, CWD and SWE anomalies**. For H2 (canopy properties → productivity), NIRv was the outcome and CIre, CCI, NDII and FRic were predictors. For H3 (climate + canopy properties → productivity), NIRv was the outcome, climate anomalies were focal predictors and the canopy properties were candidate mediators.

Pre-processing and statistical models.
To target within-plot, year-to-year effects, we formed demeaned versions of time-varying variables by subtracting each plot's mean from the annual value (Mundlak/within–between decomposition). We then applied an inverse hyperbolic sine (*asinh*) transformation, where $\text{asinh}(x) = \ln(x + \text{sqrt}(x^2+1))$. Analyzing outcomes and treatments on the *asinh* scale provides the benefits of a log transformation without the limitations that a log transformation brings with it. Like a log transformation, *asinh* compresses skewness and makes multiplicative effects more linear and homoscedastic. Unlike the log transformation, however, it has the advantage of being defined at zero and for negative values, hence being applicable to anomalies without dropping or shifting observations. Finally, we performed linear mixed models (LMMs, using REML; lme4/lmerTest R packages) on transformed anomalies, including a random intercept for plot to capture time-invariant site heterogeneity and year fixed effects (factor) to absorb space-invariant temporal shocks (Wooldridge, 2002).

Estimand and identification.
Our core estimand was the within-plot effect of a given climate anomaly (or mediator) on the outcome in the same year, net of plot-invariant differences and shared year shocks. The plot random intercept (or plot fixed effect in the TWFE check, see below) removes all time-invariant confounding among plots (e.g., persistent soils, topography, long-term management differences), while the year fixed effects absorb any Alps-wide shocks common to all plots in a given year (e.g., sensor-wide artifacts, pan-Alpine anomalies not captured by the focal driver). Identification assumes that remaining time-varying within-plot confounders are adequately controlled by the covariate set. Because canopy properties and productivity were summarized over overlapping portions of the same growing season, temporal precedence between mediator and outcome cannot be established from the annual observations alone; the corresponding coefficients are therefore interpreted as conditional associations consistent with, but not proof of, the proposed direction.

As a robustness check, and to ensure the modelling approach was coherent with estimation based on a different modelling approach, we re-estimated our core mixed-effects models using a two-way fixed-effects

(TWFE) approach. For each hypothesis, we fit linear regressions with plot and year as fixed effects to isolate within-plot and year-to-year variation (the same estimand targeted by the LMMs). We used the same variables and transformations (on the *asinh* scale) and the same set of controls, meaning that point estimates were directly comparable. Uncertainty was computed using cluster-robust standard errors at the plot level. We implemented two-way fixed-effects with fixest::feols (Imai & Kim, 2021), absorbing plot and year effects and extracting the coefficients for the same drivers and mediators as in the LMMs. The results of the TWFE models closely matched the LMM within-effects, providing support for our conclusions not depending on the modelling framework but reflecting stable, within-plot relationships (Fig.S4).

We addressed three questions with the linear mixed models. For H1 (climate → canopy properties), we fitted one LMM for each canopy property. For H2 (canopy properties → productivity), we fitted one LMM for NIRv on the candidate canopy mediators. For H3 (climate + canopy properties → productivity), we fitted one LMM for NIRv on climate anomalies and the candidate canopy mediators. Within-plot effects were coefficients for demeaned predictors (i.e., temporal anomalies on each plot) interpreted as expected change in the response when a predictor deviated from its plot mean. For moderate to large magnitudes, *asinh(y)* behaves very much like *log(y)*, so coefficients can be read approximately as semi-elasticities (percent-type changes), while when close to zero, it behaves linearly, thus preserving information where values are small.

Piecewise structural equation modeling (SEM).

To separate direct climatic pathways from indirect pathways through canopy functional properties, we linked the fitted LMMs in a piecewise SEM. For each candidate mediator, the H1 model estimated the relationship between a climate anomaly and CIre, CCI, NDII or FRic. For FRic, this pathway represents variation in within-plot spatial heterogeneity among pixel-integrated canopy states rather than alpha functional diversity. The H3 productivity model then estimated the conditional association between that canopy property and NIRv while also estimating the remaining climate coefficient after holding the included canopy properties constant (Fig. 1). Because the mediator and outcome summaries overlap within the growing season, these products quantify pathways consistent with mediation under the stated identification assumptions, rather than demonstrating strict within-season temporal succession.

For each climate driver, the direct component was the coefficient of that driver in the productivity model that included the canopy functional properties. Each indirect component was the product of the climate-to-canopy coefficient and the conditional canopy-to-NIRv coefficient. We calculated this product for each candidate mediator and summed the products to obtain the total indirect component; adding the direct coefficient yielded the total climate association. Statistical uncertainty was obtained from the common spatial block-bootstrap replicates described below, rather than from a normal approximation to the fitted coefficient covariance.

Assessment of residual spatial autocorrelation and spatial block-bootstrap inference.

Although the analyses targeted within-plot anomalies and included plot effects, spatial dependence can remain when neighbouring plots experience similar unmeasured conditions or model errors. We therefore assessed residual spatial autocorrelation for each of the four H1 models, the H2 productivity model, and the H3 productivity model. Conditional residuals were extracted at the plot-year level. To avoid mixing temporal and spatial dependence, Moran's I was calculated separately within each year for eight geographic distance classes: 0–10, 10–25, 25–50, 50–75, 75–100, 100–150, 150–200, and 200–300 km. The resulting values were then summarized across years for each model. Residual dependence was strongest at short distances and declined progressively with distance. Within 10 km, median Moran's I across years was approximately

0.46–0.48 for the NIRv models and 0.23–0.27 for CIre, CCI and NDII, whereas it was weaker for FRic. Median Moran's I was close to zero from approximately 150 km onward for all models.

Positive residual spatial autocorrelation implies that nearby plots contain partly redundant information. Model coefficients estimated from the complete dataset remain the full-data estimates of the targeted within-plot relationships, but standard model-based errors can be too small if nearby observations are treated as more independent than they are. This can produce confidence intervals that are too narrow and overly optimistic evidence that an effect differs from zero. We therefore based the final statistical inference on a non-parametric spatial block bootstrap. A primary block width of 150 km was selected because it approximately matched the distance at which residual autocorrelation had decayed to near zero, while retaining enough spatial units for resampling. We additionally evaluated 100- and 200-km blocks and translated grid origins as sensitivity analyses, to verify that conclusions were not driven by a single block width or arbitrary grid placement.

Plots were assigned to a regular square grid in the projected coordinate system used for the analyses (EPSG:3035), with cells measuring 150 × 150 km. The spatial block, rather than the individual plot, was treated as the resampling unit. In each of 1,000 bootstrap replicates, we sampled with replacement the same number of occupied blocks as in the original partition. Whenever a block was selected, all plots within that block and the complete 2017–2024 time series of each selected plot were included. Thus, neighbouring plots remained together within a resampled block, preserving short- and intermediate-distance spatial dependence. If a block was selected more than once, its observations were duplicated and assigned new bootstrap plot identifiers so that repeated copies were treated as distinct resampled clusters in the mixed model.

For every replicate, the panel dataset was reconstructed and all within-plot anomaly variables were recomputed using the same preprocessing as in the full-data analysis. The H1, H2, and H3 mixed models were then refitted with the same response variables, predictors, controls, plot effects, year effects, transformations, and estimation settings as the original models. The same spatial resample was used for all component models within a replicate. This common-resample design was important because it preserved the covariance among the estimated climate-to-canopy, canopy-to-productivity, and direct climate-to-productivity pathways. Spatial-bootstrap uncertainty was quantified from the empirical distribution of the corresponding coefficient across the 1,000 replicates. We calculated 95% percentile intervals as the 2.5th and 97.5th percentiles of the bootstrap distribution. An effect was described as supported when this interval excluded zero. We also recorded the proportions of bootstrap estimates above and below zero and calculated a two-sided sign-based bootstrap probability as twice the smaller of these two proportions. Model convergence, singularity, and the number and proportion of successful replicates were recorded for each coefficient. The bootstrap changes the estimated uncertainty and effective spatial replication, but it does not replace the coefficient estimated from the full dataset.

For the piecewise structural equation model, direct, indirect, and total effects were recalculated within each common bootstrap replicate. For a given climate driver and canopy mediator, the indirect effect was the product of the climate-to-mediator coefficient from the corresponding H1 model and the mediator-to-NIRv coefficient from the H3 model. Mediator-specific indirect effects were summed to obtain the total indirect effect, and the direct H3 climate coefficient was added to obtain the total climate effect. Confidence intervals for direct, mediator-specific indirect, summed indirect, and total effects were obtained from their empirical distributions across the 1,000 spatial resamples. Computing all path products within the same

replicate accounts jointly for spatial dependence and for covariance among the component coefficients, and replaces the original normal-approximation uncertainty propagation.

We applied the same 150-km spatial block bootstrap to all varying-coefficient models corresponding to the focal H1, H2, and H3 pathways shown in Figure 5, irrespective of whether the average pathway effect had a 95% spatial-bootstrap interval excluding zero. This allows environmental moderation to be evaluated even when the average relationship is uncertain, because a weak or unsupported average effect can mask contrasting responses across environmental contexts. For each pathway, one complete-case dataset was defined and used for both the full-data fit and every bootstrap replicate. Static moderators were standardized using means and standard deviations calculated from that full-data model sample, and these constants were kept fixed across replicates. Each resampled varying-coefficient model retained the original control variables and random-effects structure. We extracted the average within-plot slope and each driver-by-environment interaction, and calculated 95% percentile intervals from their bootstrap distributions. The mapped local effects remained full-data point estimates from the varying-coefficient models, whereas inferential statements about environmental moderation were based on the spatial-bootstrap intervals of the interaction coefficients.

Spatially explicit varying-coefficient LMMs.
To quantify how each focal within-plot pathway varied across the study area, we fitted a varying-coefficient linear mixed model in which the relevant within-plot slope was allowed to vary with static environmental moderators (see the analysis workflow Figure 3). Here, the within-plot effect of treatment on outcome is allowed to change linearly with the static environment by adding interaction terms with static moderators (here: topography, soils, climate). Each moderator is standardized (mean zero, unit variance across the training plots) to make coefficients comparable and to define a clear reference environment (here: the mean of the plots). The mixed-model backbone stays identical to the main LMMs (here: the random intercept for plot and year), meaning that the varying-coefficient model remained directly comparable to the previous models.

Spatial maps of the local effects.
After fitting a varying-coefficient model, we computed, for every pixel, the within-plot, slope of the outcome with respect to the driver. This value equals the baseline within-plot slope (evaluated at the average environment of the training plots) plus the linear adjustments that the standardized static moderators at that pixel contribute with. We retained only pixels where the standardized static moderator values fell within the range observed across training plots. This prevented including model predictions in environmental conditions that were not represented in the data. To represent parameter uncertainty in the mapped effects, we sampled from the coefficients' estimated multivariate normal distribution (using the model’s coefficient covariance), recomputed the local slope for each draw, and summarized across draws to obtain a mean effect map and a standard-deviation map. These reflect uncertainty in the estimated coefficients, conditional on the random-effects structure and the moderator rasters.

Each mapped value shows by how much the outcome **(i.e CCI, CIre, NDII or NIRv)** on the *asinh* scale changes within a plot and year, for a one-unit increase in the driver on the *asinh* scale, at the environmental conditions of the focal pixel. Including the same within-demeaned controls as described above ensured numerical comparability of average effects. The spatial variation around that average was then attributed to the static environment via the varying-coefficient terms.

S4 - Comparing with LMM and TWFE approach for modelization

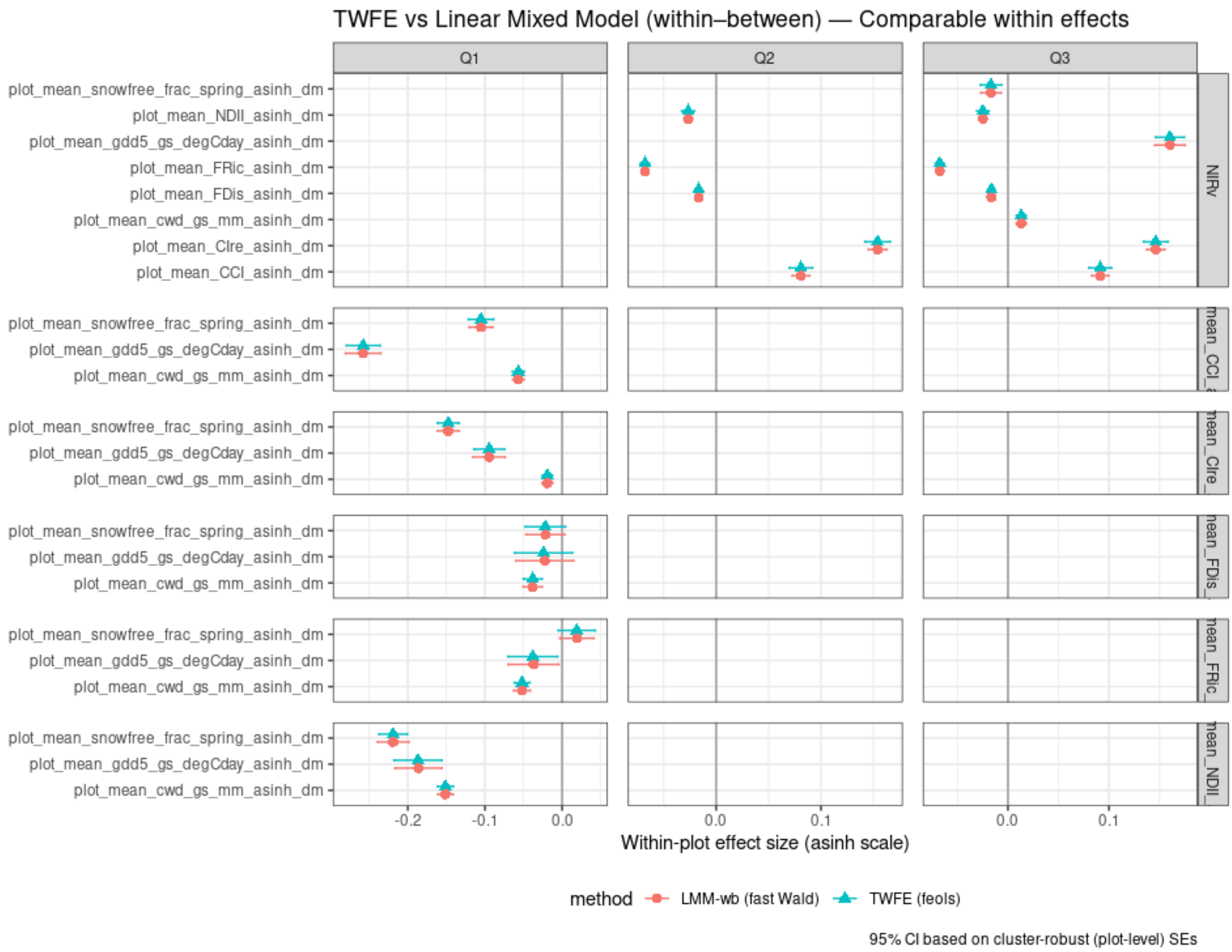

Figure S4. Comparison of within-plot coefficient estimates obtained with the demeaned linear mixed-model (LMM) and two-way fixed-effects (TWFE) approaches. Panels correspond to H1 (climate → canopy properties), H2 (canopy properties → productivity, NIRv), and H3 (climate + canopy properties → NIRv). Salmon circles show LMM estimates and teal triangles show TWFE estimates; horizontal intervals indicate the corresponding 95% confidence intervals. Both approaches use the same variables, transformations, controls and year effects and target the same within-plot estimand. The close agreement in effect magnitude and direction indicates that the main conclusions are robust to the choice of modelling framework.

Across all links (H1 climate ⟶ traits, H2 traits ⟶ NIRv, H3 climate ⟶ NIRv conditional on traits), the two-way fixed-effects (TWFE) estimates (teal triangles) and the demeaned LMM estimates (salmon circles) are virtually indistinguishable in both magnitude and sign, with overlapping 95% cluster-robust CIs throughout . No coefficient changes sign, and any differences are below the plotting resolution. This equivalence is expected because model specifications target the same within-plot parameter. TWFE with plot and year fixed effects is algebraically the within (demeaned) estimator: it removes all between-plot and common-year variation and fits OLS to the plot-demeaned regressors and outcomes. The LMM used in the main analyses is parameterized in a within–between decomposition and we report the within (demeaned) slope; with only a random intercept (no random slopes) and cluster-robust (plot-level) standard errors, the GLS/LMM coefficient on the within component coincides with the TWFE coefficient under standard regularity conditions (strict exogeneity after FE, many plots, no small-T bias in levels). Because both models use the same set of controls (year effects, same covariates) and inference is based on plot-clustered SEs, the resulting point estimates and uncertainty are effectively the same. Minor numerical differences can arise from weighting/df conventions (REML vs. OLS, Satterthwaite vs. HC/CRV variance), but they are immaterial here. For our panel of Alpine grasslands, conclusions about **climate–canopy–productivity links** do not

depend on whether we use TWFE or a demeaned LMM: both recover the same short-term, within-plot sensitivities while controlling for unobserved, time-invariant plot characteristics and common year shocks.

S5 - Decomposition of climate effects into direct, indirect, and total components

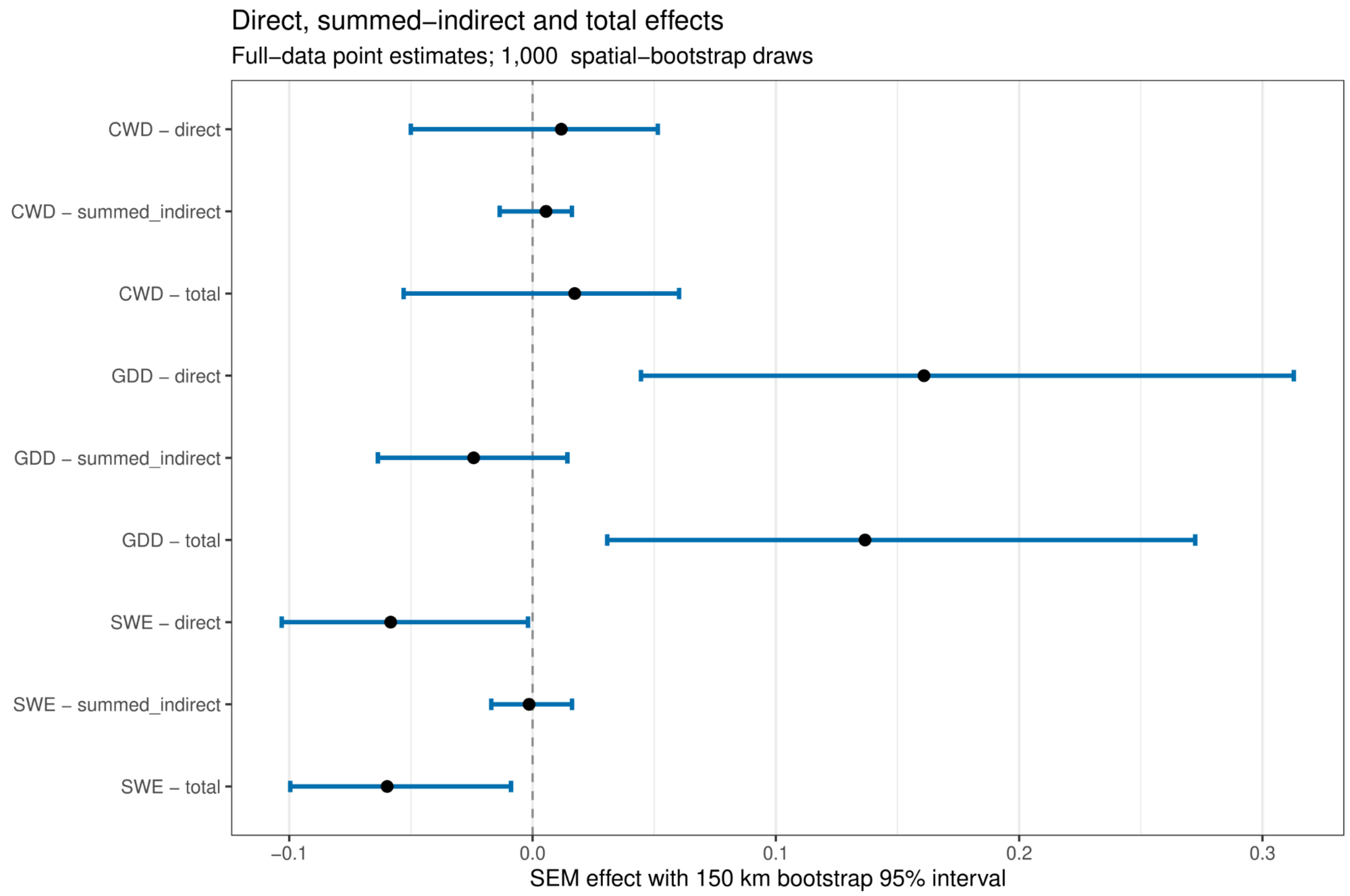

Figure S5. Direct, summed indirect, and total associations of interannual climate anomalies with vegetation productivity. Black points show the effect estimates obtained from the complete dataset. Blue horizontal intervals show the 2.5th–97.5th percentile intervals obtained from 1,000 common-resample 150-km spatial-block bootstrap replicates. Direct effects correspond to the conditional climate coefficients in the productivity model, summed indirect effects are the sum of the mediator-specific climate → canopy property → NIRv path products, and total effects are the sum of the direct and summed indirect components. Intervals overlapping zero indicate effects that were not statistically supported under the spatial-bootstrap inference.

Spatial-bootstrap inference supported positive direct and total associations of GDD with NIRv and negative direct and total associations of SWE with NIRv, whereas the summed indirect effects of both drivers had intervals overlapping zero. For CWD, the direct, summed indirect, and total associations all had intervals overlapping zero (Figure S5).

S6 - Indirect climate effects, decomposed by mediator

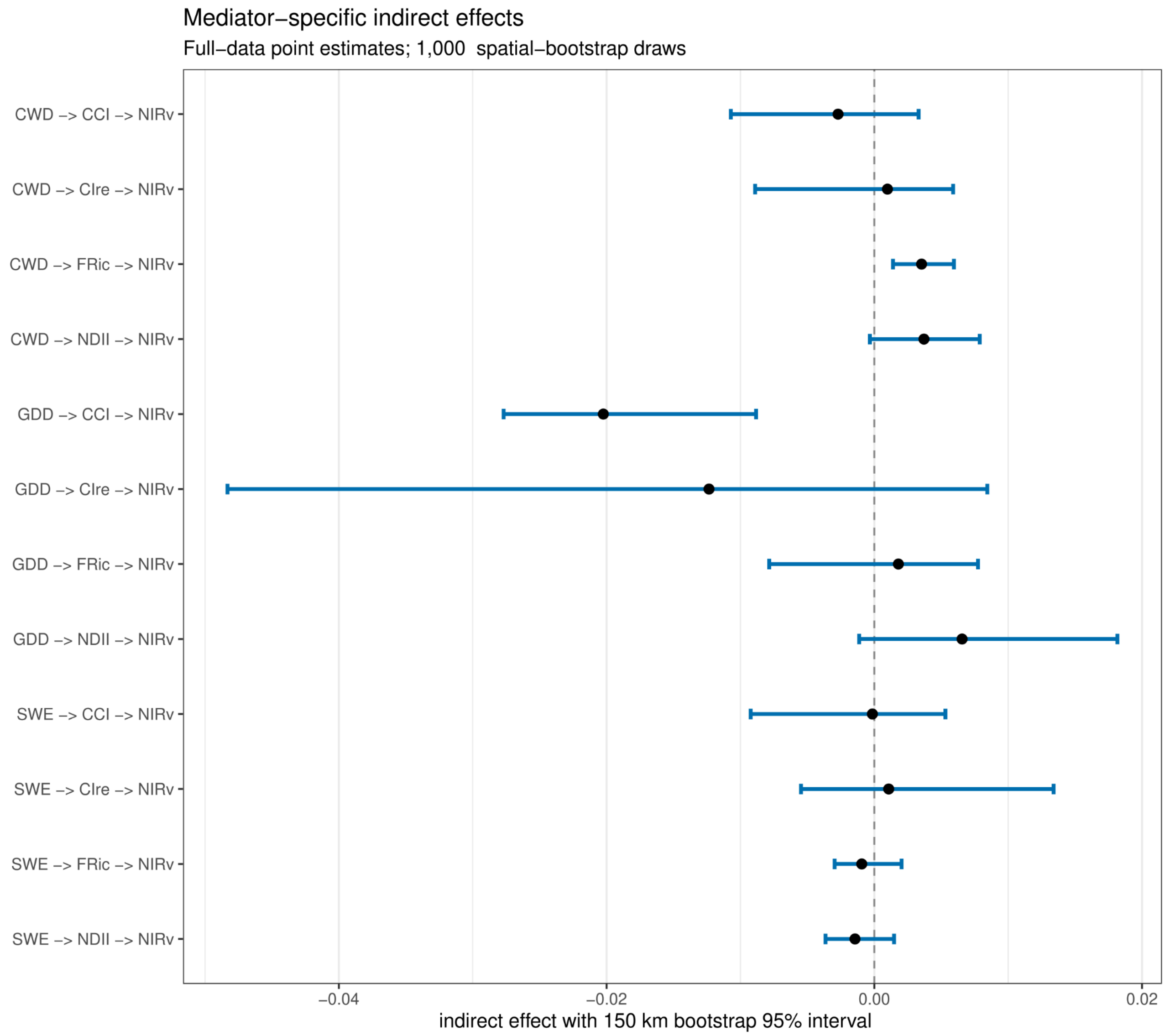

Figure S6. Mediator-specific indirect associations of interannual climate anomalies with vegetation productivity. Black points show the mediator-specific indirect effect estimates obtained from the complete dataset for each climate → canopy property → NIRv pathway. Blue horizontal intervals show the 2.5th–97.5th percentile intervals obtained from 1,000 common-resample 150-km spatial-block bootstrap replicates. These indirect effects correspond to the products of the climate → canopy-property and canopy-property → NIRv path coefficients for each mediator separately. **For FRic, the mediator denotes within-plot spectral functional heterogeneity among pixel-integrated canopy states.** Intervals overlapping zero indicate mediator-specific indirect effects that were not statistically supported under the spatial-bootstrap inference.

The mediator-specific decomposition showed that the negative GDD → CCI → NIRv pathway and the positive CWD → FRic → NIRv pathway had 95% spatial-bootstrap intervals excluding zero. The remaining mediator-specific indirect pathways, including the CWD → NDII → NIRv pathway and all SWE-mediated pathways, had intervals overlapping zero (Figure S6).

S7 - Environmental controls and mapping

GDD → NDII

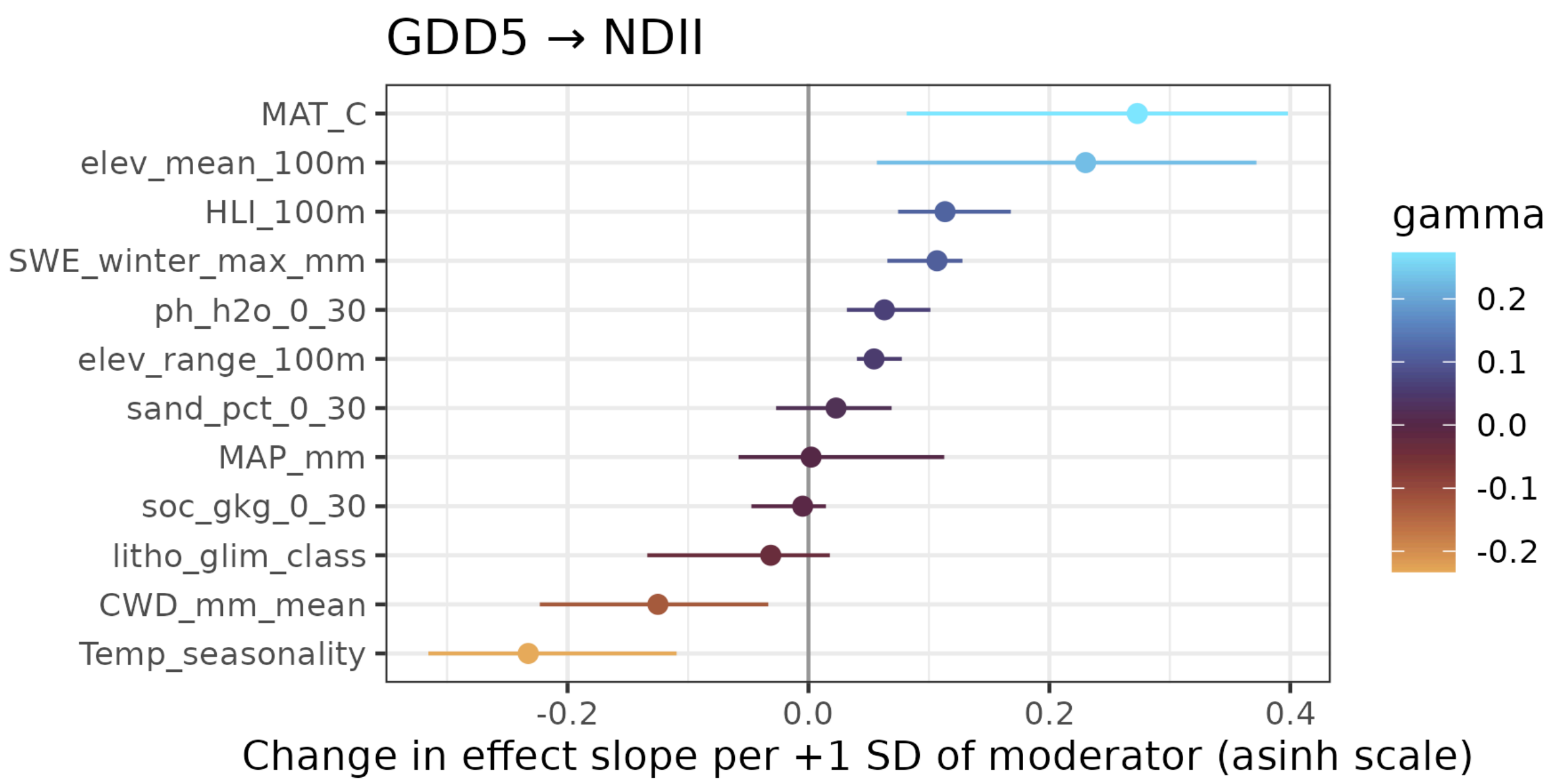

Environmental moderators of the within-plot slope GDD→NDII estimated from the varying-effect LMM. Points are environmental effect coefficients, i.e change in GDD

→NDII effect per +1 SD of the moderator (with 95% spatial-bootstrap percentile intervals).

Results description: The full-data GDD→NDII slope was negative, but its average spatial-bootstrap interval overlapped zero. Environmental context nevertheless explained 15% of plot-to-plot variation in the fitted GDD→NDII slope. Spatial-bootstrap intervals supported negative moderation by temperature seasonality and long-term CWD, indicating more negative GDD→NDII slopes in more seasonal and drier baseline climates. In contrast, MAT, mean elevation, HLI, winter SWE, soil pH, and elevation range had positive moderator effects, attenuating the negative slope. Sand fraction, MAP, SOC, and lithology had intervals overlapping zero.

Ecological interpretation: NDII proxies canopy water status. The supported negative moderation by temperature seasonality and long-term CWD is consistent with warm anomalies producing stronger canopy drying where climates are already more seasonal or water-limited. By contrast, the positive moderation by MAT, elevation, HLI, winter SWE, soil pH, and local elevation range indicates that the GDD→NDII response becomes less negative along these gradients. Because the average GDD→NDII effect itself was uncertain, these patterns are best interpreted as spatial heterogeneity in the direction and magnitude of canopy-water responses rather than as evidence for a uniformly negative warming effect across the Alps.

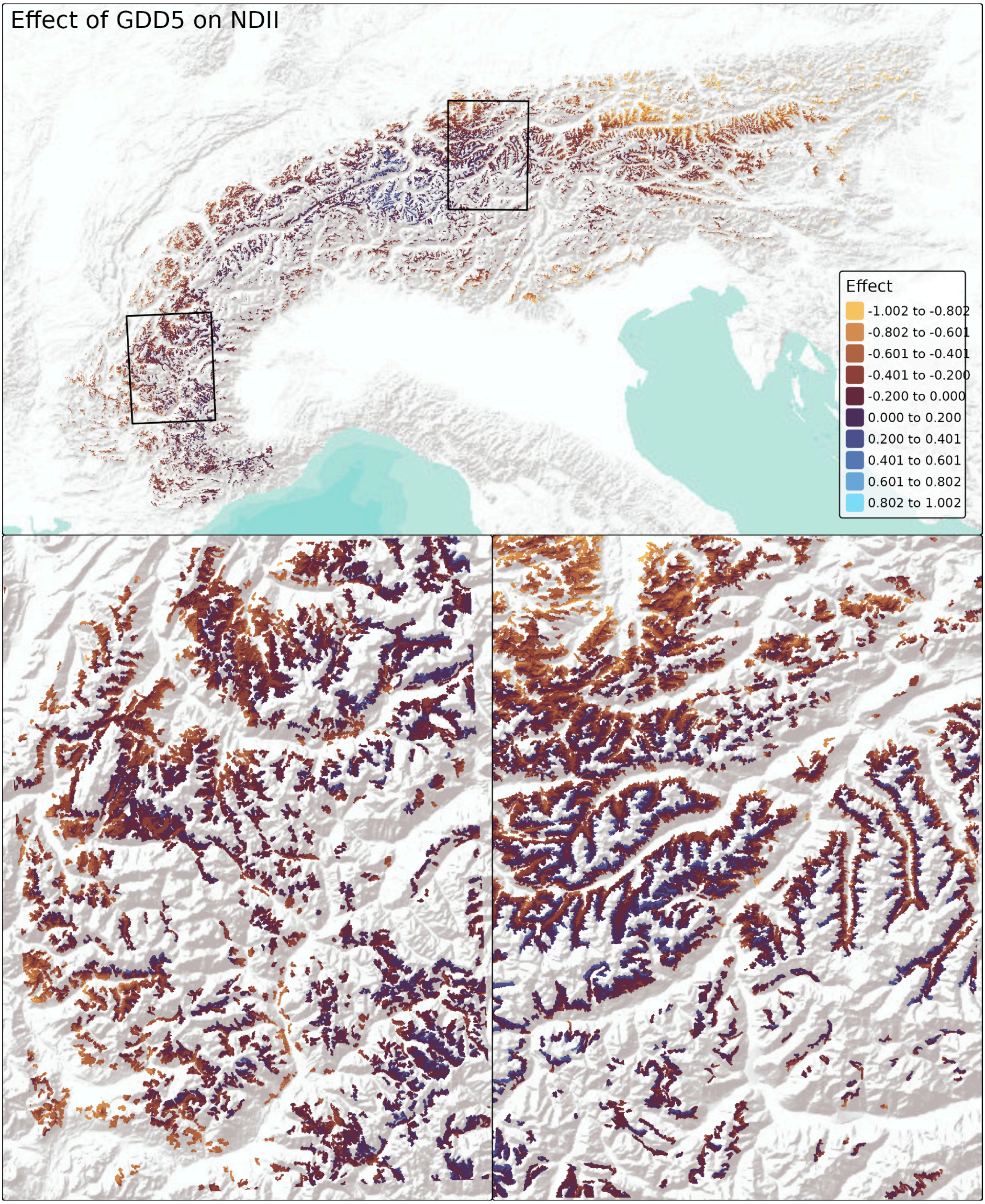

Predicted spatial distribution of the within-plot GDD→NDII effect from the varying-coefficient LMM. Colors show the magnitude and sign of the GDD→NDII slope on the asinh scale (orange = more negative; blue = more positive).

Transfer to geographic space: The fitted GDD → NDII map showed stronger negative responses in parts of the north-eastern and external Alps, whereas responses were weaker or locally close to zero in parts of the south-western and inner Alps. This broad contrast is consistent with the supported negative moderation by temperature seasonality and long-term CWD, which make GDD → NDII slopes more negative in more seasonal and drier baseline climates, and with the supported positive moderation by MAT, mean elevation, HLI, winter SWE, soil pH, and local elevation range, which attenuate the negative relationship. The insets

also show pronounced fine-scale topographic structure, with adjacent valleys, slopes, ridges, and snow-influenced areas differing in fitted effect magnitude. Because the spatial projection combines all fitted moderators, including coefficients whose spatial-bootstrap intervals overlap zero, these local contrasts are interpreted descriptively rather than attributed to unsupported individual moderators. Overall, the map illustrates substantial spatial heterogeneity in canopy-water responses to warm years rather than a uniformly negative GDD effect across the Alps.

CIre→NIRv

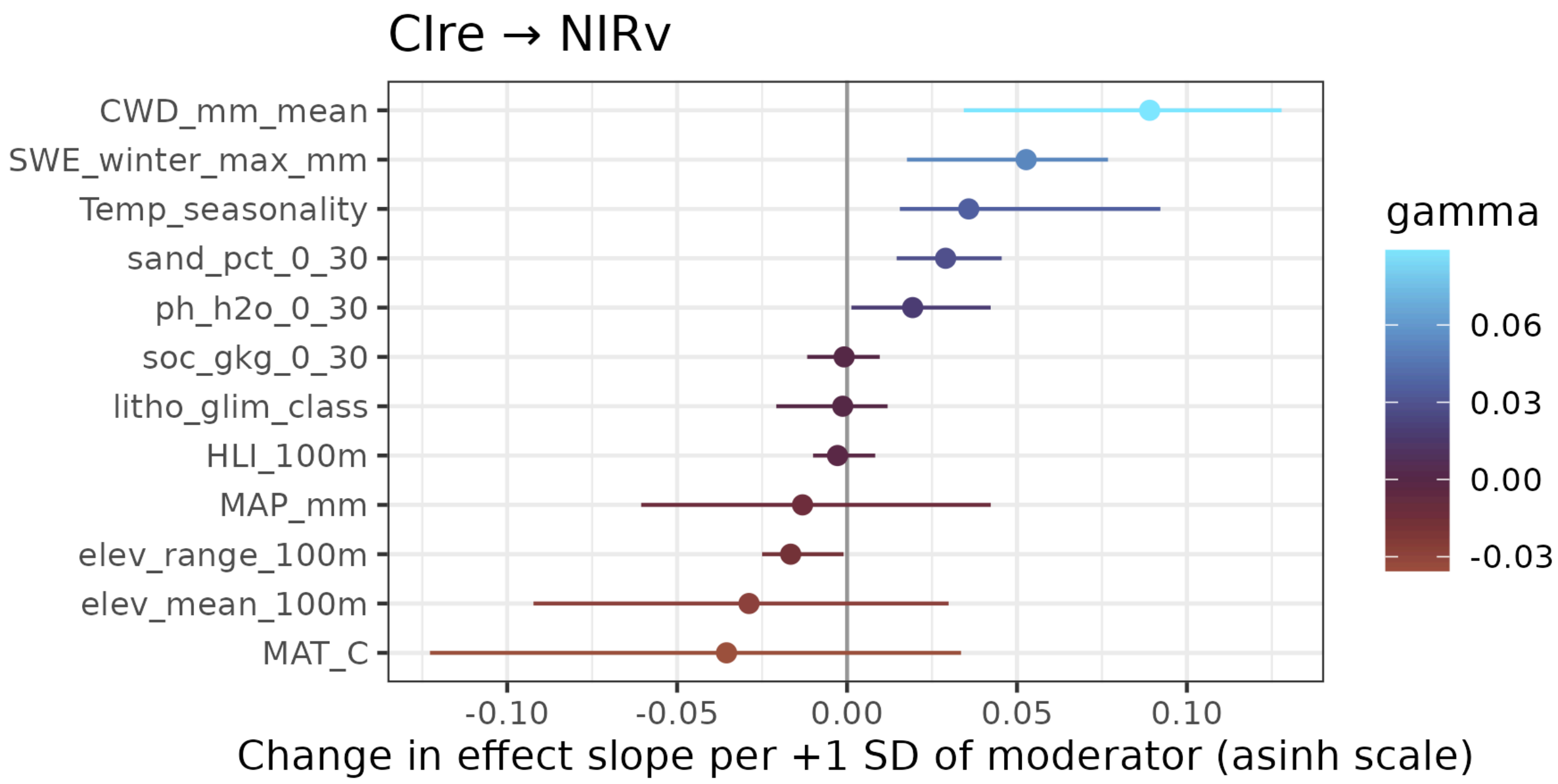

Static moderators of the within-plot effect of CIre on NIRv from the varying-coefficient LMM. Points are γ estimates (change in the CIre→NIRv slope per +1 SD of each standardized moderator) with 95% spatial-bootstrap percentile intervals. Positive γ means the CIre→NIRv coupling becomes stronger (more positive) as the moderator increases; negative γ means it becomes weaker.

Results description: Across plots and years, the CIre→NIRv slope was positive, while static environment explained only 8% of plot-to-plot variation in its strength. Spatial-bootstrap intervals supported stronger positive coupling with higher baseline CWD, winter SWE, temperature seasonality, sand fraction, and soil pH, and weaker coupling with greater local elevation range. MAP, MAT, mean elevation, HLI, SOC, and lithology had intervals overlapping zero.

Ecological interpretation: CIre tracks chlorophyll-related canopy state and potential photosynthetic capacity. The supported positive moderation by CWD, SWE, and temperature seasonality suggests that interannual increases in chlorophyll-related canopy state translate more strongly into productivity where water limitation, snow constraints, or seasonal climatic contrast are greater. Positive moderation by sand fraction and soil pH similarly indicates tighter coupling in those soil contexts, although the underlying mechanisms cannot be isolated here. Greater local elevation range weakened the coupling, consistent with fine-scale environmental heterogeneity diluting the plot-integrated CIre–NIRv relationship. Because MAP and the remaining moderators were uncertain after spatial bootstrap, we do not interpret them mechanistically.

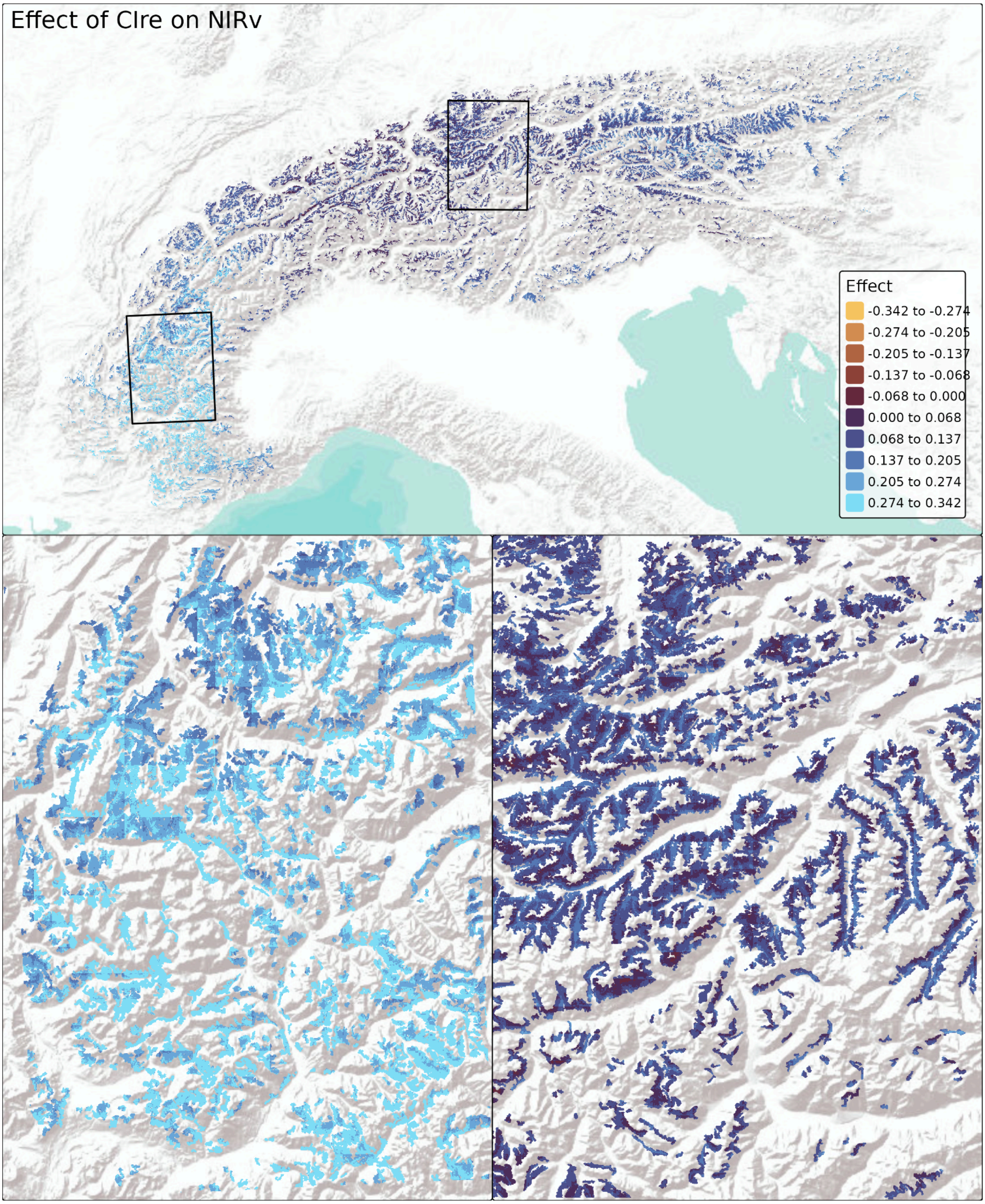

Transfer to geographic space: The map is dominated by purple and blue tones, indicating a widespread positive CIre → NIRv coupling with modest macro-scale heterogeneity. Stronger positive responses occur in parts of the south-western Alps and eastern interior ranges. These patterns are broadly consistent with the supported positive moderation by baseline CWD, winter SWE, temperature seasonality, sand fraction, and soil pH, whereas areas with greater local elevation range tend to show weaker coupling. We do not attribute spatial patterns to MAP, MAT, mean elevation, HLI, SOC, or lithology because their spatial-bootstrap intervals overlap zero.

The insets show that meso- to local-scale structure is present but not dominant. Coherent stronger-response tracts occur along some snow-influenced divides and drier interior basins, while more rugged areas often show weaker fitted coupling, consistent with the supported elevation-range moderation. The absence of a simple domain-wide spatial pattern emphasizes that the map integrates several environmental gradients simultaneously. Overall, it depicts a broadly positive CIre → NIRv relationship with localized strengthening and weakening; finer spatial contrasts are interpreted descriptively because the map combines supported and uncertain moderator coefficients.

FRic→NIRv

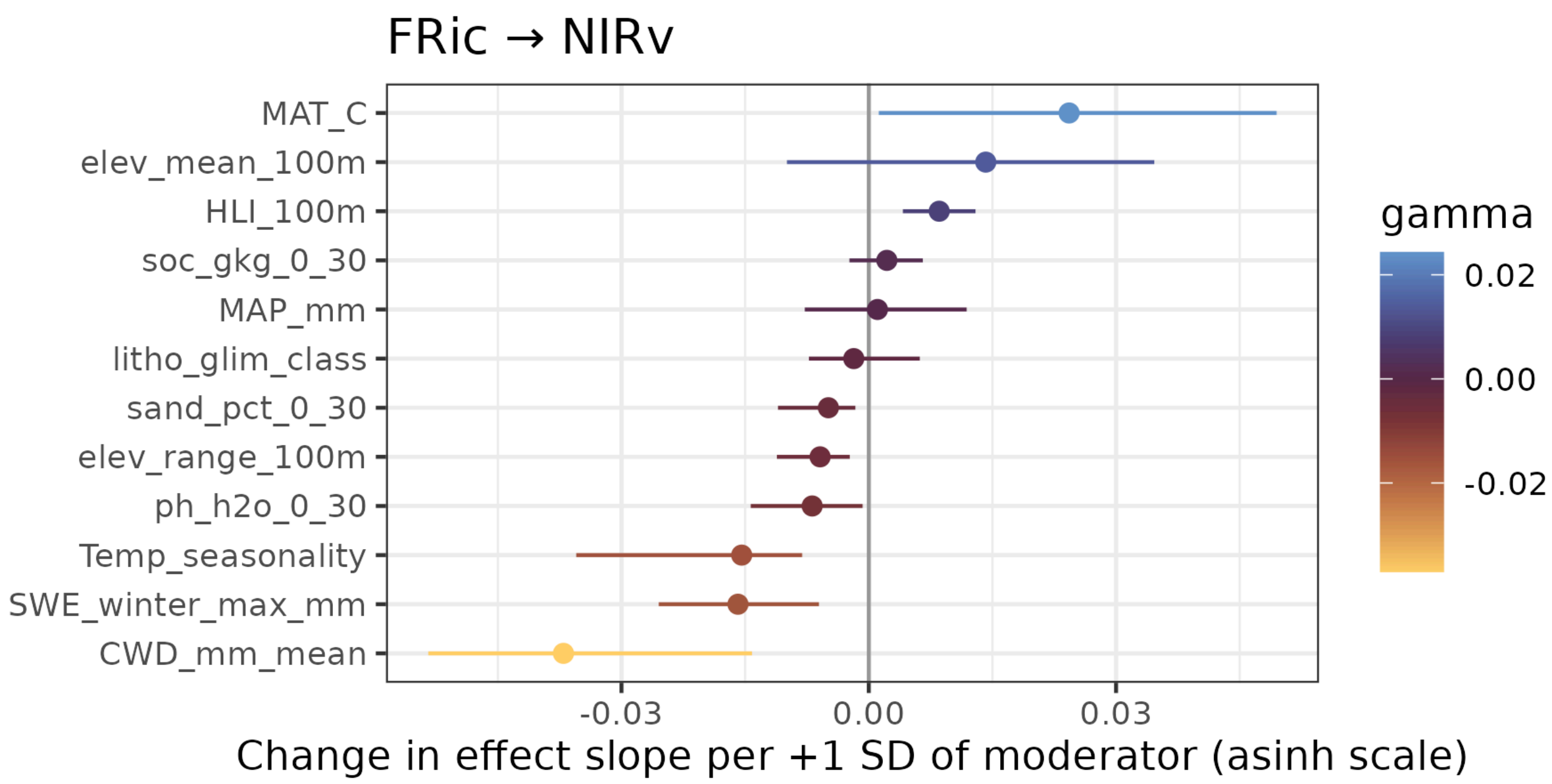

Coefficient plot. Static moderators of the within-plot FRic→NIRv slope from the varying-coefficient LMM. Points are γ estimates (change in the FRic→NIRv slope per +1 SD of each standardized moderator) with 95% spatial-bootstrap percentile intervals. Positive γ values attenuate the negative FRic→NIRv relationship (move it toward zero or positive values), whereas negative γ values strengthen the negative relationship.

Results description: Across plots and years, the FRic→NIRv slope was negative on average, with static environment explaining only 4% of plot-to-plot variation in effect strength. Spatial-bootstrap intervals supported stronger negative coupling with higher baseline CWD, winter SWE, temperature seasonality, soil pH, sand fraction, and local elevation range. In contrast, higher MAT and HLI attenuated the negative relationship. MAP, mean elevation, SOC, and lithology had intervals overlapping zero.

Ecological interpretation: **At interannual scales, increases in within-plot spectral functional heterogeneity (FRic) may reflect phenological or physiological mixing, shifts in dominance, and fine-scale spatial heterogeneity rather than immediate gains in canopy photosynthetic capacity. This FRic metric should not be interpreted as individual- or species-level alpha functional diversity.** The supported negative moderation by CWD, SWE, and temperature seasonality indicates that the negative FRic→NIRv association is strongest in drier, snowier, and more seasonally variable climatic contexts. Smaller negative moderation by soil pH, sand fraction, and local elevation range suggests that soil conditions and fine-scale heterogeneity can also sharpen this association. Positive moderation by MAT and HLI indicates that the relationship is attenuated in warmer and more highly insolated settings. These interactions modify an otherwise weak negative b-path and should not be interpreted as evidence that greater within-plot spectral heterogeneity itself causally reduces productivity.

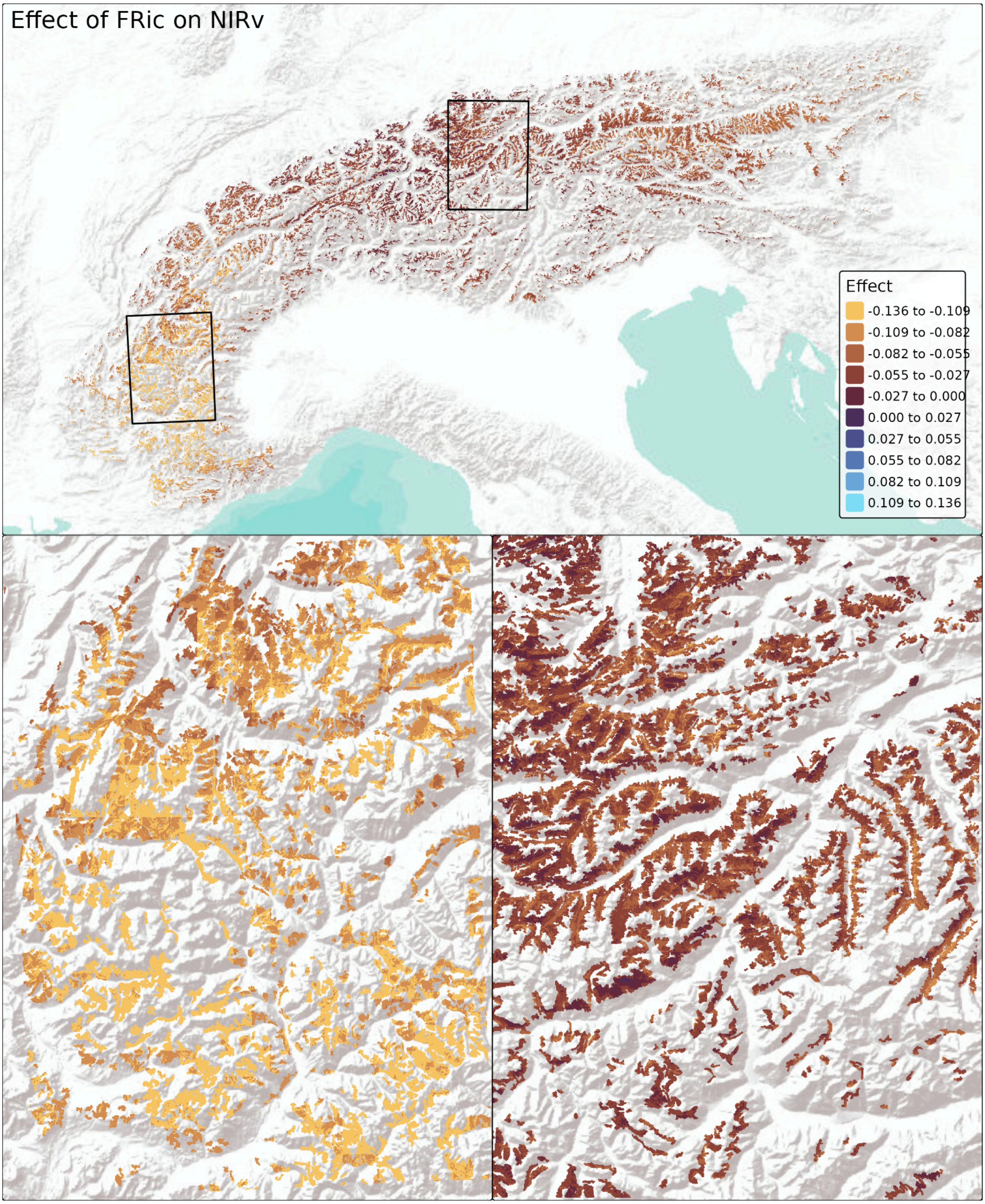

Transfer to geographic space: The map is dominated by yellow–orange tones, indicating a broadly negative FRic ⟶ NIRv relationship with modest macro-scale heterogeneity. The relationship is more negative in much of the south-western inset and weaker in parts of the central–eastern Alps. This spatial contrast is broadly consistent with the supported negative moderation by baseline CWD, winter SWE, temperature seasonality, soil pH, sand fraction, and local elevation range, which strengthen the negative association, and with the supported positive moderation by MAT and HLI, which attenuate it. At finer scales, the insets show local

contrasts between exposed ridges, shaded slopes, rugged terrain, and different soil contexts. Because the map combines all fitted moderators, including MAP, mean elevation, SOC, and lithology whose spatial-bootstrap intervals overlap zero, these local patterns are interpreted descriptively rather than assigned to unsupported individual drivers. Overall, the spatial projection illustrates where the negative FRic → NIRv association is stronger or weaker without implying that greater FRic itself causally reduces productivity.

Supplementary materials References

Ali, A. M., Darvishzadeh, R., Skidmore, A., Heurich, M., Paganini, M., Heiden, U., & Mücher, S. (2020). Evaluating prediction models for mapping canopy chlorophyll content across biomes. *Remote Sensing*, *12*(11), 1788.

Badgley, G., Field, C. B., & Berry, J. A. (2017). Canopy near-infrared reflectance and terrestrial photosynthesis. *Science Advances*, *3*(3), e1602244.

Clevers, J. G. P. W., & Gitelson, A. A. (2013). Remote estimation of crop and grass chlorophyll and nitrogen content using red-edge bands on Sentinel-2 and -3. *International Journal of Applied Earth Observation and Geoinformation: ITC Journal*, *23*, 344–351.

Croft, H., Chen, J. M., Luo, X., Bartlett, P., Chen, B., & Staebler, R. M. (2017). Leaf chlorophyll content as a proxy for leaf photosynthetic capacity. *Global Change Biology*, *23*(9), 3513–3524.

Damm, A., Paul-Limoges, E., Haghighi, E., Simmer, C., Morsdorf, F., Schneider, F. D., van der Tol, C., Migliavacca, M., & Rascher, U. (2018). Remote sensing of plant-water relations: An overview and future perspectives. *Journal of Plant Physiology*, *227*, 3–19.

Enquist, B. J., Kerkhoff, A. J., Stark, S. C., Swenson, N. G., McCarthy, M. C., & Price, C. A. (2007). A general integrative model for scaling plant growth, carbon flux, and functional trait spectra. *Nature*, *449*(7159), 218–222.

Enquist, B. J., Norberg, J., Bonser, S. P., Violle, C., Webb, C. T., Henderson, A., Sloat, L. L., & Savage, V. M. (2015). Chapter Nine - Scaling from Traits to Ecosystems: Developing a General Trait Driver Theory via Integrating Trait-Based and Metabolic Scaling Theories. In S. Pawar, G. Woodward, & A. I. Dell (Eds.), *Advances in Ecological Research* (Vol. 52, pp. 249–318). Academic Press.

Gamon, J. A., Huemmrich, K. F., Wong, C. Y. S., Ensminger, I., Garrity, S., Hollinger, D. Y., Noormets, A., & Peñuelas, J. (2016). A remotely sensed pigment index reveals photosynthetic phenology in evergreen conifers. *Proceedings of the National Academy of Sciences of the United States of America*, *113*(46), 13087–13092.

Gitelson, A. A., Keydan, G. P., & Merzlyak, M. N. (2006). Three-band model for noninvasive estimation of

chlorophyll, carotenoids, and anthocyanin contents in higher plant leaves. *Geophysical Research Letters*, *33*(11). https://doi.org/10.1029/2006gl026457

Grime, J. P. (1998). Benefits of plant diversity to ecosystems: immediate, filter and founder effects. *The Journal of Ecology*, *86*(6), 902–910.

Hardisky, M. A., Klemas, V., & Daiber, F. C. (1983). Remote sensing salt marsh biomass and stress detection. *Advances in Space Research: The Official Journal of the Committee on Space Research (COSPAR)*, *2*(8), 219–229.

Helfenstein, I. S., Schneider, F. D., Schaepman, M. E., & Morsdorf, F. (2022). Assessing biodiversity from space: Impact of spatial and spectral resolution on trait-based functional diversity. *Remote Sensing of Environment*, *275*(113024), 113024.

Imai, K., & Kim, I. S. (2021). On the use of two-way fixed effects regression models for causal inference with panel data. *Political Analysis: An Annual Publication of the Methodology Section of the American Political Science Association*, *29*(3), 405–415.

Körner, C. (2021a). *Alpine Plant Life: Functional Plant Ecology of High Mountain Ecosystems*. Springer Nature.

Körner, C. (2021b). Plant ecology at high elevations. In *Alpine Plant Life* (pp. 1–22). Springer International Publishing.

Loreau, M., & de Mazancourt, C. (2013). Biodiversity and ecosystem stability: a synthesis of underlying mechanisms. *Ecology Letters*, *16 Suppl 1*, 106–115.

Loreau, M., & Hector, A. (2001). Partitioning selection and complementarity in biodiversity experiments. *Nature*, *412*(6842), 72–76.

Rossi, C., Kneubühler, M., Schütz, M., Schaepman, M. E., Haller, R. M., & Risch, A. C. (2020). From local to regional: Functional diversity in differently managed alpine grasslands. Remote Sensing of Environment, 236, 111415.

Schneider, F. D., Morsdorf, F., Schmid, B., Petchey, O. L., Hueni, A., Schimel, D. S., & Schaepman, M. E. (2017). Mapping functional diversity from remotely sensed morphological and physiological forest traits. Nature Communications, 8(1), 1441.

Shipley, B., De Bello, F., Cornelissen, J. H. C., Laliberté, E., Laughlin, D. C., & Reich, P. B. (2016). Reinforcing

loose foundation stones in trait-based plant ecology. *Oecologia*, *180*(4), 923–931.

Stephenson, N. (1998). Actual evapotranspiration and deficit: biologically meaningful correlates of vegetation distribution across spatial scales. *Journal of Biogeography*, *25*(5), 855–870.

Violle, C., Navas, M.-L., Vile, D., Kazakou, E., Fortunel, C., Hummel, I., & Garnier, E. (2007). Let the concept of trait be functional! *Oikos* , *116*(5), 882–892.

Wipf, S., & Rixen, C. (2010). A review of snow manipulation experiments in Arctic and alpine tundra ecosystems. *Polar Research*, *29*(1). https://doi.org/10.3402/polar.v29i1.6054

Wooldridge, J. M. (2002). *Econometric Analysis of Cross Section and Panel Data*. MIT Press.

Yu, L., Liu, Z., & Li, Y. (2024). Evaluation on spatiotemporal consistency between solar-induced chlorophyll fluorescence and vegetation indices in grassland ecosystems. *PloS One*, *19*(11), e0313258.

Zhao, Y., Wu, Z., Zhao, Y., Zheng, Z., Lu, X., Sun, W., Wang, Y., & Bai, Y. (2025). Inferring scalable productivity-related grassland functional diversity in combination with in-situ leaf spectra and Sentinel-2 data. Fundamental Research, 5, 2073–2083.

Zheng, Z., Cong, N., Zhao, G., Zhao, B., Zhu, Y., Zhang, Y., Zhu, J., Zhang, T., Chen, N., Gao, J., Zhang, Y., & Sun, Y. (2024). Evident influence of water availability on the relationship between solar-induced chlorophyll fluorescence and gross primary productivity in the alpine grasslands of the Tibetan Plateau. International Journal of Applied Earth Observation and Geoinformation: ITC Journal, 129(103821), 103821.
